# Prognostic and therapeutic potential of microRNAs for fracture healing processes and non‐union fractures: A systematic review

**DOI:** 10.1002/ctm2.1161

**Published:** 2023-01-11

**Authors:** Franziska Lioba Breulmann, Luan Phelipe Hatt, Boris Schmitz, Esther Wehrle, Robert Geoff Richards, Elena Della Bella, Martin James Stoddart

**Affiliations:** ^1^ AO Research Institute Davos Davos Platz Switzerland; ^2^ Department of Orthopedic Sports Medicine Klinikum Rechts der Isar Technical University of Munich Munich Germany; ^3^ Institute for Biomechanics ETH Zürich Zurich Switzerland; ^4^ Department of Rehabilitation Sciences Faculty of Health University of Witten/Herdecke Witten Germany; ^5^ DRV Clinic Königsfeld Center for Medical Rehabilitation Ennepetal Germany; ^6^ Faculty of Medicine Medical Center‐Albert‐Ludwigs‐University of Freiburg Albert‐Ludwigs‐University of Freiburg Freiburg Germany

**Keywords:** biomarker, bone healing, fracture healing, microRNA, non‐union fractures

## Abstract

**Background:**

Approximately 10% of all bone fractures result in delayed fracture healing or non‐union; thus, the identification of biomarkers and prognostic factors is of great clinical interest. MicroRNAs (miRNAs) are known to be involved in the regulation of the bone healing process and may serve as functional markers for fracture healing.

**Aims and methods:**

This systematic review aimed to identify common miRNAs involved in fracture healing or non‐union fractures using a qualitative approach. A systematic literature search was performed with the keywords ‘miRNA and fracture healing’ and ‘miRNA and non‐union fracture’. Any original article investigating miRNAs in fracture healing or non‐union fractures was screened. Eventually, 82 studies were included in the qualitative analysis for ‘miRNA and fracture healing’, while 19 were selected for the ‘miRNA and fracture non‐union’ category.

**Results and conclusions:**

Out of 151 miRNAs, miR‐21, miR‐140 and miR‐214 were the most investigated miRNAs in fracture healing in general. miR‐31‐5p, miR‐221 and miR‐451‐5p were identified to be regulated specifically in non‐union fractures. Large heterogeneity was detected between studies investigating the role of miRNAs in fracture healing or non‐union in terms of patient population, sample types and models used. Nonetheless, our approach identified some miRNAs with the potential to serve as biomarkers for non‐union fractures, including miR‐31‐5p, miR‐221 and miR‐451‐5p. We provide a discussion of involved pathways and suggest on alignment of future research in the field.

## Graphical abstract

1. Non‐union and delayed fracture healing are major problems for patient treatment; biomarkers such as miRNA can help predicting complications.
2. A systematic review approach identified key miRNA involved in non‐union or delayed fracture healing.
3. Identification of the 6 most investigated miRNA in fracture healing with role in key pathways.
4. The use of predictive markers can lead to improved patient treatment via preventive and theranostics measures.

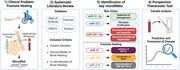

## 1 BACKGROUND

Despite surgical and treatment improvements for trauma‐related diseases, approximately 10% of fractures do not heal fully.^1^ The main traditional risk factors related to non‐union or delayed fracture healing are older age, female sex, smoking, diabetes mellitus (DM) and obesity.^2^ However, the accuracy of predictions based on these factors remains poor and may not be used to guide early interventions to prevent non‐unions.

MicroRNAs (miRNAs) are small, noncoding RNAs involved in the regulation of gene expression pathways^3^ by driving messenger RNA (mRNA) degradation and translational repression, influencing pivotal cellular processes, such as cell proliferation, differentiation, apoptosis and cell migration.^4^, ^5^, ^6^

Recently, miRNAs have been discussed as promising predictive markers since they are indicative of cellular processes and can be assessed non‐invasively in blood and other body fluids in the form of a ‘liquid biopsy’.^3^, ^4^, ^7^ For example, miRNAs are already investigated to be used as biomarkers for cancer^8^ and are involved in maintaining vasculo‐protective functions.^9^ As they are functional molecules, they also hold great promise in theranostic approaches. Until now, these miRNAs are still in the early stages of investigation and have to be validated before translation into clinical practice.

### 1.1 The process of fracture healing

For bone healing processes, two different ossification mechanisms may take place: intramembranous and endochondral ossification.^10^ During intramembranous ossification, bone regenerates directly by differentiation of mesenchymal stromal cells (MSCs) into osteoblasts. Intramembranous ossification occurs within a few days at the periosteal sites characterised by low strain and hydrostatic pressure^11^ at distal edges of the fracture site and leads to a hard callus formation.^12^ Bridging across the central fracture gap provides initial stabilisation, leading to first biomechanical functions.^13^ Subsequent differentiation of the MSCs into end‐stage osteoblasts leads to new bone formation.^14^ In contrast, endochondral ossification is a bone regeneration process in which bone heals indirectly through the formation of a cartilage intermediate.^10^, ^15^ Endochondral ossification occurs primarily in long bones such as femur, tibia or humerus, which are not rigidly fixed and therefore allow motion between the bony ends of the fracture. Cartilage formation, as the first step during endochondral ossification, occurs in less stable regions with higher strains, where no direct ossification can take place and thus occurs mostly in regions close to the fracture site.^12^ During endochondral ossification, MSCs differentiate into chondrocytes and start building a cartilaginous extracellular matrix. This produces a callus that subsequently mineralises, and the mineralised callus is remodelled into bone.

Bone repair in general is divided into three different phases: the inflammatory, the reparative or proliferative and the remodelling phases.^16^ Initially, a haematoma is formed and inflammation in the fracture region occurs. The haematoma acts as a source of signalling molecules that initialise fracture healing, including interleukins (IL‐1 and ‐6), tumour necrosis factor alpha^13^, ^17^ and growth factors, such as transforming growth factor‐β1 (TGF‐β1), fibroblast growth factors (FGFs), platelet‐derived growth factor (PDGF) and bone morphogenic proteins (BMPs).^18^, ^19^ BMPs are part of the TGF‐β superfamily, and key players in MSC proliferation and differentiation.^20^ For example, BMP‐2 directs the differentiation of cells from the periosteum or marrow cavity into a chondrogenic or osteogenic phenotype.^21^ The following reparative phase is defined by vascular remodelling and recruitment of mesenchymal progenitor cells that will differentiate into chondrocytes or osteoblasts.^16^ The differentiation of MSCs into bi‐potential osteochondral progenitor cells is initially regulated by sex determining region Y‐box 9 (*SOX9*) expression.^22^ Remodelling is dynamically regulated by the activity of osteoblasts, osteocytes and osteoclasts. During remodelling, the degradation of callus tissue by osteoclasts is followed by replacement of woven bone with lamellar bone.^23^ Including the remodelling phase, the whole fracture healing process can last up to several years.^24^

During the formation of new bone tissue, the expression of genes encoding for collagen type I and II, as well as other extracellular matrix components, including osteocalcin, osteonectin and osteopontin, change over time and marker genes can be detected in in vitro experiments and indicate either differentiation towards chondrogenesis or osteogenesis. For osteogenic differentiation, common markers are osteocalcin (*BGLAP*), osteopontin (*SPP1*), runt‐related transcription factor 2 (*RUNX2*), alkaline phosphatase (*ALPL*) and collagen type I (*COL1A1* and *COL1A2*).^25^ Well‐established chondrogenic markers are collagen type II (*COL2A1*), aggrecan core protein (*ACAN*), cartilage oligomeric matrix protein (*COMP*) and *SOX9*.^26^ In addition, a number of miRNAs have been identified to regulate central osteogenic differentiation markers.^27^ For example, miR‐9 inhibition increases mRNA levels of *RUNX2* and *BMP7* in bone tissue at the fracture site.^27^

### 1.2 The role of vascularisation, innervation and mechanical load during fracture healing

Fracture healing is supported by vascularisation, innervation and mechanical loading. The initial haematoma is a temporary matrix for the invasion of the vascular network,^28^ which provides oxygen and nutrients and removes waste, including necrotic bone tissue resorbed by osteoclasts.^29^ Here, the vascular endothelial growth factor (VEGF) signalling pathway is the principal mediator, stimulating angiogenesis, bone formation and callus mineralisation,^30^ with BMP2 promoting angiogenesis by increasing VEGF production in osteoblasts.^31^

Bone is a highly innervated tissue and the peripheral nervous system is directly involved in osteogenesis through secretion of neuropeptides, such as vasoactive intestinal peptide and calcitonin gene‐related peptide,^32^, ^33^, ^34^ which modulate osteogenic differentiation.^35^

Mechanical loading, and particularly the strain across the fracture gap, is one major determinant of the fracture healing process and influences the time for the fracture to heal, the ossification route and the stability of the newly formed bone.^36^ Mechanical forces influence the differentiation of MSCs by improving or preventing angiogenesis,^37^ as well as activating the TGF‐β/BMP pathway during the fracture healing process.^38^, ^39^, ^40^

miRNAs are also involved in the control of bone remodelling, particularly by regulating osteoclast and osteoblast differentiation and function. Changes in miRNA expression levels influence the function, apoptosis and proliferation of bone cells, and can regulate differentiation processes.^41^, ^42^

### 1.3 miRNAs and bone diseases

Bone diseases, such as osteoporosis or osteoarthritis, are a common and increasing problem in the ageing population and miRNAs have already been investigated as predictive markers for individual outcomes of bone diseases.^43^ For example, miR‐146a/b has been shown to regulate the expression of *FGF2*, which is associated with bone mineral density (BMD), the main diagnostic variable for osteoporosis.^44^ Higher levels of FGF stimulate osteoclastogenesis, which enhances bone resorption, leading to lower BMD.^44^ Of note, miR‐21, miR‐23a and miR‐24 have been found to be upregulated in the serum of patients who endured a bone fracture, and a similar miRNA profile was detected in osteoporotic bone tissue,^45^ indicating that blood miRNA levels resemble tissue miRNA composition. Today, many studies have reported on changes in miRNA expression in osteoporotic fractures in animal models (e.g., induced by bilateral ovariectomy in rodents) and investigated miRNA expression during healing. Together, these studies indicate that miR‐21 promotes early bone repair in rat models of osteoporosis and miR‐21‐3p improves the healing of osteoporotic fractures in mice.^46^, ^47^ The increasing understanding of the pivotal roles of miRNAs in time and special bone healing processes has set the stage for miRNAs as predictive markers for delayed fracture healing and non‐unions. However, current knowledge on miRNAs in bone healing originates from diverse clinical populations, a wide range of different tissues and cell populations and a multitude of animal and cell models, with partly conflicting findings.

### 1.4 Objective

This review aimed to summarise and structure the findings from clinical populations, animals and cell models to identify miRNAs with the potential to be used as biomarkers to monitor the fracture healing process. Several studies have already investigated the role of miRNAs in fracture healing processes to find potential biomarkers for non‐union fractures or fracture healing in general. This review aims to detect and discuss the unknown main regulators and highlight promising miRNAs that have the potential to be used for clinical diagnosis and treatment.^48^, ^49^ An advantage of miRNAs as biomarkers is that they can be detected in biofluids, and they can be analysed in blood samples by using a small amount of blood. They are very specific, as they can directly be connected to signalling pathways and their role in target gene regulation can be assessed.

## 2 METHODS

A systematic review was used to identify common miRNAs involved in fracture healing or non‐union followed by a qualitative analysis. We screened for all miRNAs that were validated as involved in the fracture healing process and possible biomarkers for non‐union fractures. All included studies had to either (1) screen patient samples (which we defined as Type 1 Study) or (2) implement an animal model (Type 2 Study), as those studies have a high translational potential. Studies that only focused on the in vitro validation of miRNAs during chondrogenesis and osteogenesis were not selected for further qualitative analysis, as they are lack validation on a higher translational or clinical model.

### 2.1 Literature search and inclusion criteria

A systematic review (CRD42022344974) in accordance with the PRISMA guidelines^50^ and following the suggestions for reporting on qualitative summaries was performed.^51^, ^52^ Literature search was conducted using PubMed, Web of Science, EBSCO and Scopus, including variations and combinations of the following keywords: ‘microRNAs and fracture healing’ and ‘microRNAs and fracture non‐union’. Any original article investigating miRNAs in fracture healing or non‐union fractures was eligible for inclusion. Specific inclusion criteria were as follows: (1) studies investigating miRNAs in patient samples, (2) studies investigating miRNAs in animal models of bone healing and (3) studies investigating miRNAs in in vitro models of bone healing. Studies that (1) reported only on in vitro analysis, (2) only focused on small interfering RNA/long noncoding RNA, (3) were not available in English (full text), (4) were not available as full‐text or (5) retracted articles were excluded from further analysis. Conference abstracts and grey literature were not included. All records published until 28 February 2022 were eligible for inclusion.

### 2.2 Study selection, data extraction and aggregation

Data were extracted by two reviewers (Franziska Lioba Breulmann and Luan Phelipe Hatt), and tables were created including information on first author, year of publication, number of patients included/animals analysed, type of intervention, underlying diseases, differentially expressed miRNAs, miRNA analysis method (sequencing, microarray, quantitative polymerase chain reaction [qPCR]), clinical screening, type of in vitro experiment, animal fracture model and cell type. Strand information (‐3p/‐5p) was not included in the selection process since some studies indicated identical regulation independent of strands and strand information is not always provided by the authors. Direction of miRNA regulation was extracted as indicated by the authors (i.e., if statistical significance was reported). In the case of imprecise, uncommon, unclear/conflicting or missing descriptions of methods, or participants, studies were excluded.

### 2.3 Grouping of studies and synthesis

To provide a structured qualitative summary, studies were grouped into two main categories: (1) fracture healing and (2) non‐union fractures. The validity of the reported findings was assessed using categories: clinical population, animal model and cell model. Studies that were found in both literature searches were included in only one of the two main categories according to the main subject of the study. For example, some studies were found in the general search for fracture healing but included non‐union fracture patients or non‐union animal models.^53^ The certainty of the evidence was addressed using an evaluation of how directly the included studies addressed the planned question/applied methodology (measurement validity), the number of studies and the consistency of effects across studies. The risk of bias of the studies was not assessed since only studies investigating patient populations followed by animal/cell model validation were included. We qualitatively analysed the included studies to evaluate which miRNAs have already been validated to be involved in fracture healing and non‐union and which miRNAs are most promising as biomarkers for healing progress.

## 3 RESULTS

### 3.1 Literature search

**FIGURE 1:**
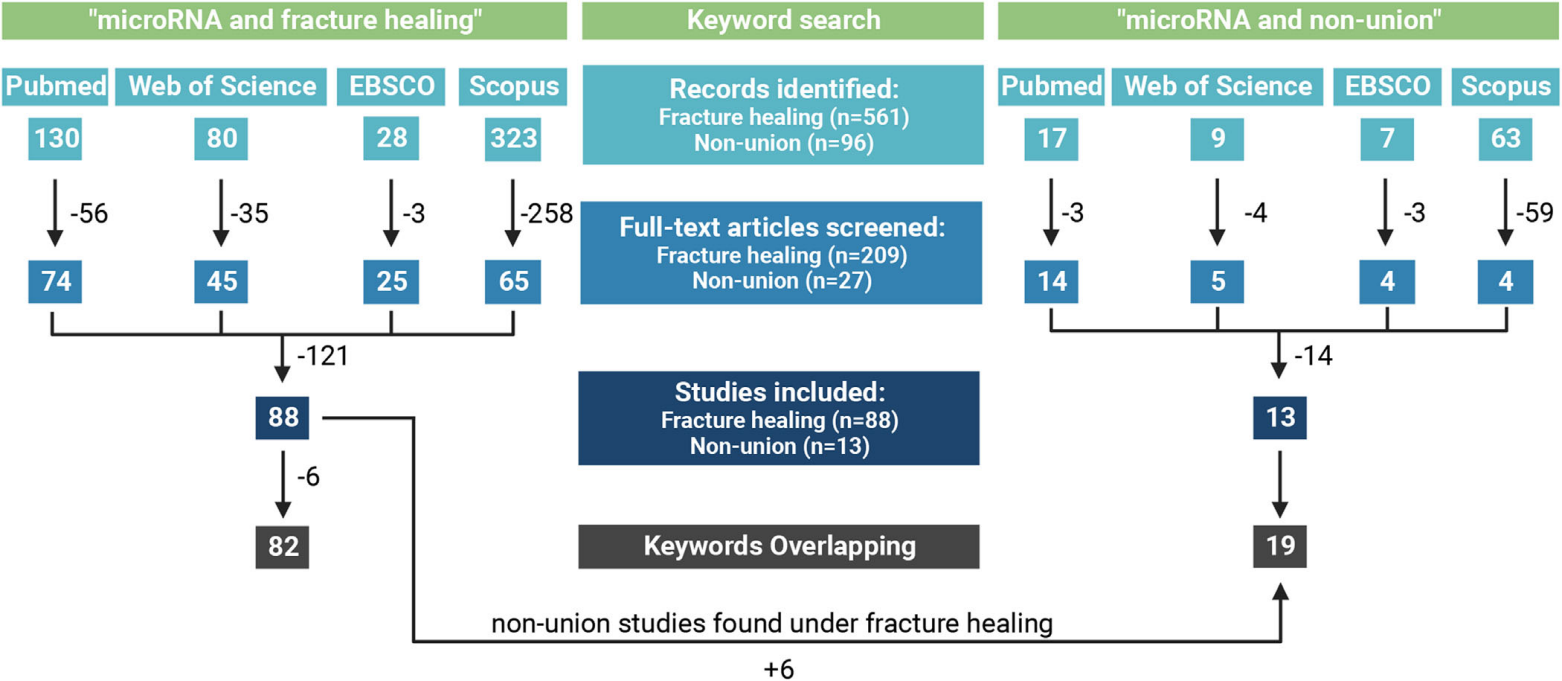
Literature search review process according to the PRISMA criteria. Reviews or record duplicates were excluded from analyses. Six records were excluded after screening of full‐text articles according to following criteria: reported only on in vitro analysis, only focused on small interfering RNA (siRNA)/long noncoding RNA (lncRNA) and not available in English (full text) or retracted articles. The included studies are summarised in Table 1, 2 and 3. In summary, 82 studies were included in the ‘microRNA and fracture healing’ analysis, while 19 studies were selected for the ‘microRNA and non‐union’ screening.

Figure 1 summarises the literature search process. In brief, the search ‘microRNAs and fracture healing’ resulted in the following: *n* = 130 records on PubMed, *n* = 80 on Web of Science, *n* = 28 on EBSCO and *n* = 323 on Scopus. On PubMed, 74 full‐text articles were screened, 45 on Web of Science, 25 on EBSCO and 65 on Scopus. From this search, 88 full‐text articles were identified as fulfilling the selection criteria. However, six full texts found using the keywords ‘microRNA and fracture healing’ were categorised in the non‐union group, as they focused on screening in non‐union fracture patients or investigating a non‐union model. In summary, a total of 82 full‐text articles were included in the fracture healing group (Table 1).

**TABLE 1:** MicroRNAs (miRNAs) regulated in fracture healing

Author (year)	miRNA	Clinical screening (patient groups [number])	In vitro cell model (cell type: conditions)	Animal model (type of model; treatment l [number of replicates])	Animal model follow‐up by X‐ray/μCT ([timepoint; analysis])	miRNA analysis (cells/tissue [timepoint]; method)	Cell line/type
Murata, K. (2014)^64^	miR‐92a ↓	Bone fracture, days 0 and 7, 14, 21 after surgery [*n* = 26]	Osteoblasts: transfection of antimiR‐92a or control LNA	Mouse fracture model: transverse mid‐diaphysis fracture [NA]	X‐ray [NA; callus formation, bridging of fracture gap, callus volume]; μCT [NA; vascularity of the fracture callus]	RNA from cells, bone tissue and blood [NA]; qPCR	Human; mouse
Seeliger, C. (2014)^45^	miR‐21 (↑), ‐23‐3p (↑), ‐24‐3p (↑), ‐25‐3p (↑), ‐27a‐3p (↑), ‐100‐5p (↑), ‐122a‐5p (↑), ‐124a‐3p (↑), ‐125b‐5p (↑), ‐148a‐3p (↑), ‐223‐3p (↑) (in osteoporotic fractures)	Hip fracture (osteoporotic vs. non‐osteoporotic): bone tissues collected during implantation of total endoprosthesis [*n* = 20]				RNA from serum samples and bone tissue [collected during surgery]; qPCR; microarray	Human
Sun, Y. (2015)^101^	miR‐21 ↑		BMSCs: transfection with pre‐miR‐21, osteogenic differentiation	Rat: closed femur fracture; injection of BMSCs overexpressing miR‐21/ctr. into the fracture site [*n* = 18 in total]	X‐ray [days 0 and 7 after surgery]; μCT [5 weeks after fracture; low‐ and high‐density mineralised tissues, mineralised callus formation, BV/TV]	RNA from cells [3 and 7 days after induction]; qPCR	Rat (BMSCs)
Li, Y. (2015)^102^	miR‐26a ↑		BMSCs: osteogenic differentiation, transfection with miR‐26a mimics or NC	Osteoporotic mouse model: ovariectomy/sham operation; subcutaneous implantation of MSCs transfected with miR‐26a mimic or NC into dorsal pocket [NA]	NA; μCT [8 weeks after ovariectomy; BV/TV, trabecular bone formation]	RNA from cells [days 0, 2, 7, 14 after transfection]; qPCR	Mouse (C57BL/6J)
Weilner, S. (2015)^66^	miR‐10a‐5p (↑), ‐10b‐5p (↑), ‐22‐3p (↑), ‐133b (↓), ‐328‐3p (↓), let‐7g‐5p (↓)	Fracture patients: post‐menopausal patients with osteoporotic femoral neck fracture [*n* = 37 in total, 19 fracture patients]	ASCs: transfection with miRNA, osteogenic differentiation of transfected ASCs			RNA from serum, adipose tissue, and cells [serum within 14 days after surgery]; qPCR; miRNA screening (175 miRNAs)	Human (ASCs)
Yuan, H.F. (2015)^72^	miR‐181c‐3p (↑), ‐34a‐3p (↑), ‐146a‐5p (↑), ‐187‐3p (↑), 181a‐3p (↑), ‐30c‐1‐3p (↑), ‐650 (↑), ‐3653 (↑), ‐4444 (↑), ‐11273e (↑), ‐99a‐3p (↑), ‐3064‐5p (↑), ‐212‐3p (↓), ‐212‐5p (↓), ‐132‐3p (↓), ‐629‐3p (↓)	Patients with hip osteonecrosis receiving total hip arthroplasty [*n* = 9]; femoral neck fracture patients (control group) [*n* = 6]				RNA from tissue samples [collected during surgery]; qPCR; microarray	Human
Furuta, T. (2016)^91^	miR‐4532 (↑), ‐125b‐5p (↑), ‐4516 (↑), ‐338‐3p (↑), ‐548a (↑)		MSCs: exosomes isolated from MSC	Mouse fracture model: transverse femoral shaft fracture [*n* = 77], conditioned‐medium exosomes were injected on the fracture site of CD9 –/– mice and WT mice [*n* = 10/group]	X‐ray [0, 1, 2, 4, 6 weeks after fracture]; μCT [0, 1, 2, 4, 6 weeks after fracture; BMD of the femur shaft, callus bridging on the cortices]	RNA from cells and exosomes [NA]; miRNA expression assay	Mouse (C57BL/6, CD9 –/– and WT); human (MSCs)
Lee, W.Y. (2016)^92^	miR‐29‐3p ↑		BMSCs: osteogenic differentiation, transfection with pmiR29b/pTeton/Sonovue or pHygro/pTeton/Sonovue	Mice femoral fracture model: midshaft femur fracture, injection of pHygro/pTeton (control group), S‐miR‐29b‐3p or repeated R‐miR‐29b‐3p at week 2 or weeks 2 and 3 after surgery [NA]	X ray [after surgery, weekly until 6 weeks; position of fixative and fracture line callus width and area]; μCT [NA; distinguish low‐density tissues from high‐density tissues by BMD of callus, BV + TV]; [NA]	RNA from cells [day 3 after osteogenic induction]; qPCR	Mouse (BMSCs)
He, B. (2016)^82^	53 differentially expressed miRNAs identified to impair fracture healing at 2‐ or 4‐week post‐fracture; miR‐494 ↓		C3H10T1/2 cells: transfection with miR‐494 mimics, anti‐miR‐494 or inactive control; chondrogenic differentiation	Mouse fracture model: transverse femoral shaft fracture [NA]	NA; μCT [0, 2, 4 weeks after fracture; BV/TV, bone mineral content as indicators of callus mineralisation]	Total RNA from callus tissues and cells [0, 2, 4 weeks after fracture]; qPCR; microarray	Mouse (C57BL/6J osteoblasts; C3H10T1/2 cells)
Hadjiargyrou, M. (2016)^103^	miR‐140‐3p (↑), ‐21a‐5p (↑), ‐142a‐3p (↓), ‐494‐3p (↓)			Mouse fracture model: femur fracture [*n* = 3/timepoint]		RNA from callus tissue samples [days 1, 3, 5, 7, 11, 14 after surgery]; qPCR; microarray (922 miRNAs)	Mouse (C57BL/6)
Li, K.‐C. (2016)^104^	miR‐140 (↓), ‐214 (↓)		BMSCS: transduction with BV vectors; OVX‐BMSCs and mock‐transduced BMSCs: osteoinduced and co‐cultured with osteoinduced cells	Rat osteoporotic bone defect model: femur fracture [NA]	NA; μCT [2 and 4 weeks after surgery; BV/TV, BMD, trabecular thickness, trabecular number and distance between trabeculae]	RNA from cells [NA]; qPCR	Rat (BMSCs)
Huang, J. (2016)^105^	miR‐429 ↑		Osteoblasts: mimic the effect of hypoxia by adding CoCl2	Mouse fracture model: injection of lentivirus containing miR‐429 subcutaneous in the region of the local fracture [*n* = 8/group]	X‐ray [NA, healing parameters]; NA	RNA from tissues and cells [NA]; qPCR	Mouse (MC3T3‐E1 cells)
Zou, L. (2017)^57^	miR‐124‐3p ↑	Patients with metaphyseal fracture of distal tibia [*n* = 195; *n* = 62 for blood samples]	OS‐732 cells: transfection with scramble control, miR‐132 mimics, BMP6 siRNA, miR‐124‐3p inhibitor			RNA from cells and blood samples [NA]; qPCR	Human (OS‐732 cells)
Li, Q.S. (2017)^63^	miR‐214‐5p ↑	Fracture patients: intra‐articular hand fracture [*n* = 17] and intra‐articular calcaneal fracture [*n* = 11]; blood samples collected on days 7, 14, and 21 after surgery	Osteoblasts: transfection with ASO‐miR‐214‐5p, ASO‐NC, miR‐214‐5p and pcDNA 3.1‐COL4A1 and its vector control			RNA from cells and blood samples [days 7, 14 and 21 after surgery]; qPCR	Human; mouse (MC3T3‐E1 cells)
Tu, M. (2017)^93^	miR‐142‐5p ↑		Osteoblasts: transfection with agomiR‐142‐5p, antagomiR‐142‐5p and their NC for 21 days	Mouse fracture model: mid‐diaphysis femur fracture; injection for 4 weeks of agomiR‐142‐5p/agomiR‐NC/PBS [NA]	X‐ray [day 28 after surgery; determine fracture union by bridging callus]; NA	RNA from blood samples [4 weeks after first injection] and from cells and calluses [days 0, 7, 14, 21 and 28]; qPCR	Mouse (C57BL/6, MC3T3‐E1)
Li, K.C. (2017)^106^	mir‐214 ↓		ASCs: transduction with BV‐vectors, osteoinduction; ASCs and BMSCs (from OVX or sham‐operated rats): co‐culture and osteoinduction for 15 days	Osteoporotic rat model: ovariectomy/sham operation; ASCs harvested from inguinal fat pads; BMSCs isolated from limb of rats; femur fracture and implantation of ASCs/gelatin construct [NA]	NA; μCT [2 and 5 weeks after implantation; volume of interest, BV/TV, BMD, Tb.Th, Tb.N and Tb.Sp]	RNA from cells [after 15 days of co‐culture]; qPCR	Rat
Tian, Z. (2017)^83^	miR‐495 ↑		Osteoblasts: transfection with miR‐495 mimics and miRNA‐NC; cells exposed to miRC, miR‐495 and anti‐miR‐495 for 24 h and apoptosis analysed	Mouse fracture model: drill‐hole injury at the femur; injection of miNC/anti‐miR‐495 for 21 days [*n* = 8]	NA; μCT [21 days after surgery; BV/TV, Tb.Th, Tb.N]	RNA [NA]; qPCR; microarray	Mouse (calvaria osteoblasts)
Yao, C. (2018)^107^	miR‐185 ↓		Osteoblasts: transfection with miR‐185 mimics, miR‐185 inhibitors + siPTH	Mouse femur fracture model: sacrificed after fracture to take the middle of the femur and osteoblasts isolated	X‐ray [directly after surgery]; μCT	RNA [NA]; qPCR	Mouse (C57BL/6)
Shi, L. (2018)^94^	miR‐218 ↑		Osteoblasts: transfection with lentiviral miR‐218; BMSCs: transfection with miR‐218 antisense and miR‐NC; BMSCs: osteogenic differentiation	Mouse femoral fracture model: mid‐shaft fracture; scramble/LV‐miR‐218 injection into the fracture site [*n* = 12/group]	X‐ray [fracture day, 2 and 4 weeks after surgery; bone fracture monitoring], μCT [NA; bone mineralisation, BV, TV, BV/TV]	RNA from LV‐miR‐218 and scramble infected cells; at days 3, 7, 14 after OIM induction	Mouse (C57BL/6, BMSCs)
Zhang, S.Y. (2018)^59^	miR‐203 ↑	Fragility fracture patients [*n* = 75]: hand fractures [*n* = 40] and intra‐articular fractures [*n* = 35]	Osteoblasts: transfection with plasmids			RNA on plasma samples of patients [days 1, 7 14 and 21 after fracture]	Human (hFOB1.19 cells)
Wang, F. (2018)^60^	miR‐488 ↓ (in osteoporotic fracture patients)	Patients with osteoporosis: blood samples [NA]	Osteoblasts: transfection with miR‐NC, miR‐488 mimics, mimics + si‐Dickkopf1			RNA from cells and from patient blood samples [NA]; qPCR	Mouse (MC3T3‐E1 cells); human
Teng, J.‐W. (2018)^113^	miR‐214 ↓			Mouse fracture model: tibial fracture; injection of PBS (control group), agomiR negative control or agomiR‐214‐3p at days 0, 7, 14 and 21 after fracture [*n* = 90, 30/group]	X‐ray [days 7 and 28 after surgery; location of the fracture, fracture types, density and size of the callus, state of fracture lines]; NA	RNA from callus tissues or tissues in the middle tibia [days 7, 14, 21 and 28 after modelling]; qPCR	Mouse (C57BL/6)
Liu, H. (2018)^84^	miR‐148 ↑		HEK293T: NC, transfection with miR‐148a agomir, miR‐148a antagomir, IGF1, miR‐148a agomir + IGF1	Rat fracture model: midshaft femur fracture; injection of miR‐148a agomir or IGF1, NC (DMEM injection), miR‐148a‐agomir + IGF1 for 6 weeks [*n* = 10/group]	X‐ray [after removal of the internal fixatives; BMD]; NA	RNA from cells [NA]; qPCR; microarray	Human (HEK293T cells); rat (BMSCs)
Sun, M.‐H. (2018)^114^	miR‐106 ↑		BMSCs: transfection with miR‐106a scramble, miR‐106a inhibitor and negative control	Rat fracture model: tibial fracture; three groups: bone fracture group, negative control (miR‐106a scramble BMSCs), miR‐106a inhibitor BMSCs [*n* = 60 in total]		RNA from blood samples and tissue [6 weeks after surgery]; qPCR	Rat (BMSCs)
Takahara, S. (2018)^85^	miR‐140‐3p (↓), ‐181a‐1‐3p (↑), ‐140‐5p ↓ (in DM group), ‐210‐3p (↑), ‐222‐3p (↑) (DM vs. control)			Femur fracture model: DM and control group; closed femoral shaft fracture in both groups [*n* = 116 in total; callus tissues on days 5, 7, 11, 14, 21 and 28 with *n* = 6/group and timepoint]	X‐ray [days 14, 21 and 28 after surgery; evaluation on cortices and their callus formation to evaluate fracture healing]; NA	RNA from callus samples [days 5, 7, 11, 14, 21 and 28 after surgery]; qPCR; microarray [on samples of days 5 and 11 after surgery]	Rat
Ge, J.‐B. (2018)^165^	miR‐374b ↑		MSCs: osteogenic induction, transfection with miR‐374b inhibitor	Mouse fracture model: tibial fracture model; after days 4, 8, 12, 16, 20 and 24 bone tissue of the tibia was collected [NA]		RNA from cells [after 7 days of osteogenic induction]; qPCR	Mouse (MSCs)
Silva, A.M. (2018)^86^	miR‐122‐5p (↑), let‐7d‐5p (↑), let‐7a‐5p (↑), let‐7e‐5p (↑), ‐466b‐2‐3p (↑), let‐7f‐5p (↑), ‐215 (↑), let‐7b‐5p (↑), let‐7c‐5p (↑), ‐21‐5p (↑); ‐3557‐3p (↓), ‐3543 (↓), ‐672‐3p (↓), ‐505‐3p (↓), ‐500‐5p (↓), ‐380‐5p (↓), ‐433‐3p (↓), ‐532‐3p (↓), ‐429 (↓), ‐3593‐3p (↓)		MSCs: differentiate into osteogenic, chondrogenic and adipogenic lineages	Critical size defect model in rats: cylindrical defect in femur, no defect created; sacrificed on days 3 and 14 after surgery; blood samples collected [*n* = 6/group, 3/timepoint per group]		RNA from plasma samples and splenocytes [days 3 and 14 after surgery]; qPCR; microarray	Rat
Sun, Y. (2019)^54^	miR‐16‐5p ↓ (in traumatic brain injury patients)	Normal group, fracture group, fracture + TBI [*n* = 20 total]	Osteoblast cell line: transfection with agomiR‐16‐5p, agomiR‐NC, antagomiR‐16‐5p, antagomiR‐NC	Mouse femoral fracture model: agomiR‐16‐5p or antagomiR‐16‐5p injected into the fracture site [*n* = 20 in total]; TBI concomitant to the fracture [*n* = 20]	X‐ray [NA]; μCT [segmentation, 3D morphometry, density, distance parameters, BV/TV, cortical thickness]	miRNA from callus of the fracture site [day 14 and 21 post‐operation]; qPCR	Human; mouse (MC3T3‐E1 cells)
Cui, Y. (2019)^108^	miR‐124 ↓		EPC; BMM: transfection with miR‐124 mimic or miR‐NC	Mouse femur fracture model; intravenous injection of BMMs or BMMs in combination with EPC‐derived exosomes [*n* = 20/group]	NA	RNA from BMM or bone tissue [NA]; qPCR	Mouse (BMM)
Deng, J. (2019)^27^	miR‐9 ↓			Rat femoral fracture model; mid‐femoral transverse fracture; intrathecally injection of miR‐inhibitor or miR‐NC; sham group for 8 weeks after fracture	X‐ray [NA; BMD]; NA	RNA from tissue of the fracture site [NA]; qPCR	Rat
Liu, Y. (2019)^109^	miR‐21 ↑			rat femoral fracture model; injection of PBS, antagomiR‐21, antagomiR‐NC once a week for 6 weeks [*n* = 10/group]	X‐ray [days 7 and 9 after fracture; callus growth, internal fixation position, fracture line healing and fracture alignment]; NA	RNA from callus tissues [NA]	Rat
Wang, C. (2019)^110^	miR‐1856 ↑			Mouse fracture model; model group (without treatment), injection of NC, siRNA‐SMAD6, miR‐186 mimics, miR‐186 inhibitor, miR‐186 inhibitor + siRNA‐SMAD6 [*n* = 105 in total]	X‐ray [day 0]; μCT [days 14, 28 and 42 after surgery; BV, BV/TV, BMD]	RNA from callus tissues and cells [NA]; qPCR	Mouse (C57/BL)
Zhou, L.‐G. (2019)^111^	miR‐214 ↓			Osteoporosis rat model: ovariectomy in rats, intraperitoneally injection of PBS, antagomiR‐NC or antagomiR‐214‐3p, transverse femur fracture (ex situ) [*n* = 30]	X‐ray [after surgery, days 7 and 42; internal fixation position, porosis, fracture line healing]; μCT	RNA from callus tissues [NA]; qPCR	Rat
Lang, Y. (2019)^95^	miR‐25 ↑			Rat fracture model: middle femoral fracture, intraperitoneally injection of PBS, mimics NC or miR‐25 mimics [*n* = 45 in total]	X‐ray [1 and 7 weeks after surgery; femoral fracture healing and callus formation]; μCT	RNA from callus tissues [2 weeks after surgery]; qPCR	Rat
Sheng, J. (2019)^115^	miR‐21 ↓			Rabbit fracture model: fracture group without treatment, intramuscularly injection of penicillin sodium treatment or miR‐21 siRNA (for 7 days) for 5 days twice a day [*n* = 15 in total]	X‐ray [1–20 days after surgery; bone tissue, time of bony callus formation, fracture healing]; NA	RNA from bone tissues [NA]; qPCR	Rabbit
Liu, Q.‐P. (2019)^48^	miR‐140‐3p ↑			Rat fracture model: transverse tibial fracture; intraperitoneally injection of PBS, miR‐140‐3p mimics, mimics NC, ASO‐miR‐140‐3p, ASO‐NC; for 6 weeks [*n* = 50, 10/group]	X‐ray [day 49; observe fracture healing, location of internal fixation, formation of callus and healing of fracture line]; NA	RNA from callus tissues [49 days after fracture]; qPCR	Rat
Janko, M. (2019)^166^	miR‐92a (↓), ‐335‐5p (↓)		BMCs: transfected with scrambled RNA, anti‐miR‐92A, ‐335, ‐92A and ‐355 or control anti‐miR; seeded on scaffolds; placed in femoral large bone defect	Rat fracture model: femoral fracture; BMC transplantation into bone defect [*n* = 16/group]	NA; μCT [8 weeks after surgery; BMD]	RNA from cells [after transfection; 1 and 8 weeks after surgery]; qPCR	Rat (BMCs)
Mi, B. (2019)^116^	miR‐7223‐5p ↓		MC3T3‐E1: transfection with agomiR‐7223‐5p, antagomiR‐7223‐5p, CircRNA AFF4, linear AFF4, siRNA PIK3R1	Mouse fracture model: transverse femoral fracture; injection of PBS, agomiR‐7223‐5p and plasmid CircRNA AFF4 into the fracture site on days 0, 4 and 7 [NA]	NA; μCT [NA; BV/TV, BMD]	RNA from cells or callus samples [NA]; qPCR	Mouse (MC3T3‐E1 cells)
Li, D. (2019)^80^	miR‐138‐5p ↓		BMSCs: transfection with miR‐138‐5p mimic or inhibitor	Rat femoral fracture model: femoral shaft fracture, examined by weeks 1 and 3 after surgery [*n* = 32 in total, 4/group, and timepoint]	X‐ray [NA; callus formation, bony remodelling, and implants degradation]; μCT [NA; determine implant degradation and fracture healing]	RNA from cells and tissue samples [NA]; mRNA sequencing; qPCR	Rat
Dietz, C. (2019)^70^	miR‐223‐5p ↑	Fracture patients: upper‐limb fracture or surgery, healthy controls [*n* = 20], patients with painful diabetic polyneuropathy [*n* = 158]; blood samples after overnight fasting for exosome isolation				RNA from blood [NA]; qPCR	Human
Xiong, Y. (2019)^73^	miR‐26a‐5p ↑	Fracture patients, fracture patients + TBI, patients without fracture [*n* = 6/group]	Osteoblasts: transfection with agomiR‐26a‐5p, agomiR‐NC, antagomiR‐26a‐5p, antagomiR‐NC	Mouse fracture model: mid‐diaphysis femoral fracture; fracture with concomitant TBI [*n* = 6]; blood collection on days 1 and 3 after surgery; injection of PBS (control group), agomiR‐26a‐5p, antagomiR‐26a‐5p at the fracture site on days 1, 3 and 7 after surgery [*n* = 18 in total]	NA; μCT [NA; Tb.N, BV/TV, average cortical thickness, cortical area fraction, cortical bone area, Tb.Sp, Tb.Th, total cross‐sectional area and BMD]	RNA from callus samples and cells [NA]; qPCR	Human; mouse (C57BL/6J, MC3T3‐E1 cells)
Li, X. (2019)^74^	miR‐342‐5p ↓	Fracture and healthy patients: hand fracture [*n* = 20], intra‐articular calcaneal fracture [*n* = 16], healthy controls [*n* = 20]	Osteoblasts: osteogenic differentiation, transfection with miR‐342‐5p mimics, scramble miRNA (control), miR‐342‐5p inhibitor and NC, si‐Bmp7			RNA from blood and cells [days 7, 14, 21 and 28 after surgery]	Human; mouse (MC3T3‐E1 cells)
Akkouch, A. (2019)^117^	miR‐200c ↑		hBMSCs: transfection with plasmid encoding miR‐200c, osteogenic differentiation	Mouse model: creation of transgenic mice with PMIS‐miR‐200c; calvaria defect; defect filled with collagen, plug loaded with different treatments: no treatment, only collagen, pDNA encoding EV, plasmid with miR‐200c at 1, 10 and 50 μg [NA]	NA; μCT [NA; assess craniofacial shape and abnormal growth, new bone formation analysis, BMD, BV/TV]	RNA from cells and rat explants [NA]; qPCR	Human (BMSCs); mouse
Liu, H. (2019)^118^	miR‐34a ↑		BMSCs: osteoblastic differentiation, irradiation with 0, 2, 4 and 6 Gy of X‐ray radiation, transfection with miR‐34a mimics and NC, miR‐34a inhibitor, inhibitor control, siRNA targeting Notch1 mRNA or NC	Rat osteogenesis model: BMSCs irradiated and transfected with miR‐34a mimics, mimics control and miR‐34a inhibitor, inhibitor control is subcutaneous transplanted; rat tibial defect model: tibia irradiated, bone defect conducted within 3 weeks after irradiation, newly formed bone of the defect area used for miR‐34a expression analysis; injection of agomiR‐34a and antagomiR‐34a into tibial defect [*n* = 18]	NA; μCT [8 weeks post‐implantation of miRs; bone regeneration evaluated, BV/TV]	RNA from cells and from newly formed bone tissue [2, 4 and 8 weeks after surgery]; qPCR	Rat (BMSCs)
Liu, W. (2020)^87^	miR‐126 ↑		HUVECs: exosomal and Hypo‐Exos uptake; HucMSCs: infected with LV2 vector containing miR‐126 inhibitor	Mouse femoral fracture model; mid‐diaphyseal fracture; transplantation of Exos or Hypo‐Exos to the fracture gap [*n* = 8/group]	X‐ray [7 days after fracture; callus formation], μCT [NA; vascularity at the cortical bone]	RNA from cells and exosomes [12 and 24 h], qPCR, microarray for Exos and Hypo‐Exos	Human (MSCs, HUVEC); mouse
Xu, T. (2020)^49^	miR‐128‐3p ↓		MSCs: treated with exosome‐depleted FBS; MSCs: osteogenic differentiation; transfection with miR‐128‐3p mimics and inhibitor	Rat femoral fracture model: injection of PBS, young‐Exos or aged‐Exos [*n* = 12/group]	NA; μCT [2, 3 and 4 weeks after surgery; 3D structure, mineralised callus volume, BV/TV]	RNA from cells and exosomes [7 and 14 days after treatment]; from callus of the fracture site [14, 21, 28 days post‐operation]; qPCR; microarray analysis	Rat (MSCs)
Xin, Z. (2020)^55^	miR‐214 ↓	Fragility fracture [*n* = 35]: hip fracture [*n* = 15], proximal humeral fracture [*n* = 10], distal radius fracture [*n* = 10]	Osteoblast cell line: transfection with miR‐214 mimics, mimics NC, miR‐214 AMO and AMO NC			RNA from bone tissues and blood [days 1, 7, 14, 21]; qPCR	Human; mouse (MC3T3‐E1 cells)
Xie, W. (2020)^56^	miR‐328‐3p ↓	Fragility fracture [*n* = 80], healthy patients [*n* = 40]	Osteoblasts: transfection with miR‐328 mimics or miR‐NC			RNA from blood [days 7, 14, 21] after surgery and osteoblasts [48 h after transfection]; qPCR	Human (hFOB1.19 cells)
Chen, L. (2020)^119^	miR‐701‐3p ↓		Osteoblasts: transfection with agomiR‐701‐3p, agomiR‐NC, antagomiR‐701‐3p, antagomiR‐NC, siRNA‐NC, silncRNA KCNQ1OT1	Mouse fracture model; femoral fracture; injection of PBS, siRNA‐NC, silncRNA KCNQ1oT1, agomiR‐701‐3p or antagomiR‐701‐3p on days 0, 4 and 7 after operation [*n* = 60]	X‐ray [days 0, 7, 14 and 21 after surgery; NA]; μCT [days 7, 14 and 21 after surgery; BV/TV, BMD]	RNA from cells [NA]; qPCR	Mouse (C57BL/6J, MC3T3‐E1 cells)
Mi, B. (2020)^88^	miR‐7212‐5p ↓		Osteoblast precursor cells: transfection with antagomiR‐7212‐5p, antagomiR‐NC, agomiR‐7212‐5p, agomiR‐NC and siRNA of METTL3 and FGFR3	Mouse femoral fracture model; transverse shaft fracture; injection of PBS, plasmid METTL3 and agomiR‐7212‐5p in the fracture site on days 0, 4 and 7 after surgery [*n* = 120]	NA; μCT [days 0, 4 and 7 after surgery; BV/TV, BMD]	RNA from cells or callus samples [NA]; qRT‐PCR; microarray on callus samples [days 0, 3, 5, 7, 10 and 14]	Mouse (C57BL/6J, MC3T3‐E1 cells)
Pan, L.‐X. (2020)^58^	miR‐19a‐3p ↑	Fracture patients with femoral neck fracture [*n* = 40]	osteoblasts: transfection for 48 h with si‐HAGLR			RNA from cells [NA]; qPCR	Human; mouse (MC3T3‐E1 cells)
Jiao, J. (2020)^120^	miR‐140‐5p ↑		BMM: cell transfection with miR‐140‐5p mimic	Mouse fracture model: injection of miR‐140‐5p in model group and fracture model group, negative control group for both [*n* = 8/group]	NA; μCT [NA; BMD, BV, TV, BV + TV]	RNA from cells and from callus tissues [6 weeks after surgery]; qPCR	Mouse (C3H10T1/2 cells)
Jiang, Y. (2020)^96^	miR‐25 ↑		MSCs and MC3T3‐E1C: co‐culture; BMSCs: transfection with FAMmiR‐25	Mouse fracture model: femur shaft fracture, PBS or exosomes injected into the fracture site [NA]	X‐ray [days 0, 4 and 7 after fracture; fracture site examination]; NA	RNA from callus tissues and cells [NA]; qPCR	Mouse (MSCs, MC3T3‐E1 cells)
Xiong, Y. (2020)^77^	miR‐5106 ↑		Macrophages: differentiation initiated by treating BMDMs with PBS or IL‐4; BMSCs: transfection with plasmid‐NC, plasmid‐SIK2 and plasmid‐SIK3	Mouse femoral fracture model: injection of PBS or M1D‐Exos, M2D‐Exos or M2D‐Exos with antagomiR‐5106 on days 1, 3 and 7 after fracture [NA]	X‐ray [days 7, 14 and 21 after fracture]; μCT [days 14 and 21 after surgery; BV, TV, BV/TV, BMD]	miRNA from callus samples [NA]; qPCR; microarray; RNA sequencing from M1 and M2 macrophages	Mouse (C57BL/6J)
Feng, L. (2020)^121^	miR‐378 ↓		MSCs: osteogenic differentiation, adipogenesis and chondrogenesis	Mouse fracture model: transverse femoral fracture, WT, and miR‐378‐mice [*n* = 10/group], injection of sh‐NC or sh‐miR‐378 into miR‐378 TG mice [*n* = 20]	X‐ray [directly after surgery, weekly; confirm fracture, fracture healing condition] weekly, μCT [NA; reconstruction of low and high BMD by BMD, BV, TV, BV/TV]	RNA from cells [NA]; qPCR	Mouse (miR‐378 TG); human (HEK293 cells, hBMSCs)
Hou, Y. (2020)^89^	miR‐92b ↑		MSCs: osteogenic differentiation and de‐differentiation, transfection with miR‐92b antagomir or negative control	In vivo ectopic bone formation: MSCs or miR‐92b overexpressing MSCs seeded in subcutaneous pocket; rat fracture model: mid‐femoral transverse fracture; injection of De‐Os‐MSCs or normal MSCs into fracture sites 7 days after surgery [*n* = 10]	X‐ray [3 and 8 weeks after surgery; monitor fracture healing]; μCT [3 and 8 weeks after surgery; reconstruct low‐ and high‐density mineralised tissues; bone mineralised callus, callus formation]; NA	RNA from cells [days 3, 10 after culturing]; qPCR, microarray analysis	Rat (MSCs)
Jiang, C. (2020)^97^	miR‐222 ↓		BMSCs: incubation with anti‐CD34, anti‐CD45, anti‐CH‐29 and anti‐CD44 antibodies for 30 min; transfection with miR‐222 mimic, miR‐222 inhibitor or non‐functional NC; after 24 h: transfection with TIMP‐3 stealth select RNAi or stealth RNAi NC with miR‐222 inhibitor	Rat fracture model: mid‐diaphyseal femoral fracture; intraperitoneally injection of streptozotocin for 1 week to induce diabetes, or injection of buffer (control group) [NA]		RNA from tissue samples or cells [NA]; qPCR	Rat (BMSCs)
Sun, X. (2020)^46^	miR‐21 ↑		BMSCs: isolated from osteoporotic OVX rats	Osteoporotic bone defect model in rats: tibial fracture; injection into the defects of CMCs/n (miR‐21), CMCs/n (miR‐NC), saline [NA]	NA; μCT [NA; new formed cancellous bone, BV/TV, Tb.Th, Tb.N]		Rat
Zhang, X. (2020)^47^	miR‐22‐3p ↑		BMSCs: transfection with si‐NC, si‐FTO, mimic‐NC, miR‐22‐3p, inhibitor‐NC and miR‐22‐3p inhibitor; osteogenic differentiation; isolation of EVs from supernatant of BMSCs; incubation of BMSCs with EVs for 24 h	Osteoporotic mouse model: bilateral ovariectomy, sham group [*n* = 12]; injection of PBS, BMSC‐EV, BMSC‐EV/inhibitor‐NC, BMSC‐EV/miR‐22‐3p inhibitor, miR‐22‐3p inhibitor + dimethyl sulphoxide, miR‐22‐3p inhibitor + LY294002, BMSC‐EV/inhibitor‐NC + DMSO, BMSC‐EV/inhibitor‐NC + LY294002 [*n* = 120 in total, 12/group]		RNA from cells [NA]; qPCR	Human (BMSCs); mouse
Wang, J.‐G. (2020)^167^	miR‐1 ↓		BMSCs: transfection with miR‐1 siRNA and pcDNA miR‐1 plasmids	Rat fracture model: tibial fracture [*n* = 10]		RNA from bone and blood samples [6 weeks after surgery]; qPCR	Rat (BMSCs)
Xiong, Y. (2020)^98^	miR‐7025‐5p ↓		Osteoblasts: transfection with agomiR‐7025‐5p, agomiR‐NC, antagomiR‐7025‐5p, antagomiR‐NC	Mouse fracture model: mid‐diaphysis femur fracture; injection of Cy3‐labelled agomiR‐7025‐5p on days 0, 4, 7, 10 and 14; injection of IL‐10/agomiR‐7025‐5p on the fracture site on days 1, 3 and 7 after surgery [NA]	X‐ray [days 7, 14 and 21 post‐surgery; NA]; μCT [NA; BV/TV]; NA	RNA from cells [NA]; qPCR	Mouse (C57BL/6J, MC3T3‐E1 cells)
Zarecki, P. (2020)^71^	miR‐375 (↑), ‐532‐3p (↑), ‐19b‐3p (↑), ‐152‐3p (↑), ‐23a‐3p (↑), ‐335‐5p (↑), ‐21‐5p (↑)	Post‐menopausal women [*n* = 126 in total]: sample collection from healthy controls [*n* = 42], patients with low BMD and no fracture [*n* = 39]; patients with vertebral fractures and low BMD without treatment against osteoporosis [*n* = 26], patients with vertebral fractures and low BMD receiving a treatment for osteoporosis [*n* = 19]				RNA from serum samples [after overnight fasting]; qPCR	Human
Strauss, F.J. (2020)^128^	miR‐21‐5p ↓			Tooth extraction model: miR‐21 knockout mice and littermates (WT) [*n* = 9/group]; tooth extracted and euthanised 14 days after surgery	NA; μCT [after euthanisation/14 days after surgery; BV/TV, thickness of buccal bone plate]		Mouse (C57BL/6J)
Zheng, K. (2021)^61^	miR‐193‐3p ↑	Fragility fracture patients [*n* = 70]: hand fracture [*n* = 30]; intra‐articular fracture [*n* = 40]; blood samples collected after trauma on days 0, 7, 14 and 21	Osteoblasts: transfection with miR‐193a‐3p mimic, miR‐193a‐3p inhibitor or NC, siRNA against PTEN			RNA [NA]; qPCR	Human; mouse (MC3T3‐E1 cells)
Ji, X. (2021)^62^	mRNA‐497‐5p ↓	Bone fracture patients [*n* = 80]: intraarticular fracture [*n* = 40], hand fracture [*n* = 40]	Osteoblasts: transfection with si‐PVT1, si‐NC, miR‐497‐5p mimic or inhibitor, mimic‐NC or inhibitor‐NC			RNA from cells and plasma samples [NA]; qPCR	Human (HOB1.19 cells)
Huang, Y. (2021)^99^	miR‐19b ↑		BMSCs: transduction with miR‐19b mimic/inhibitor, WWP1 overexpression plasmid, Smurf2 overexpression plasmid, KLF5 overexpression plasmid, β‐catenin overexpression plasmid, shRNA targeting KLF5 or NC	Mouse fracture model: transverse femoral shaft fracture; sham/control group, injection of miR‐19b agomiR, oe‐WWP1, oe‐Smurf2, sh‐KLF5 or sh‐β‐catenin alone in exosomes or in combination at the fracture site [*n* = 10/group]	X‐ray [0 and 4 weeks after fracture; fracture healing, callus growth and wound area]; NA	RNA from cells [48 h after transduction]	Human (BMSCs); mouse (C57)
Bourgery, M. (2021)^78^	Total of 54 out of 806 miRNAs were differentially expressed during fracture healing (25 characteristic to bone, 29 characteristics to cartilage tissue homeostasis)			Mouse fracture model: medullary tibia fracture [*n* = 75]	X‐ray [NA]; NA	RNA from callus and other tissue samples [days 5, 7, 10, 14 and 25 after fracture]; qPCR; sequencing	Mouse (C57B1/6N)
Yu, H. (2021)^122^	miR‐136‐5p ↑		Osteoblasts: osteogenic differentiation, transfection with miR‐136 mimic or inhibitor, NC; BMSCs: transfected with Cy3‐miR‐136‐5p	Mouse fracture model: femur fracture; sham group (control), model group (fractured without treatment), injection of agomiR‐NC, miR‐136‐5p, NC‐mimic‐Exos + overexpression‐NC; NC‐mimic‐Exos + oe‐DKK1, miR‐136‐5p mimic‐Exos + oe NC and miR‐136‐5p mimic‐Exos + oe‐DKK1 [*n* = 10/group] [NA]	NA; μCT [NA; to verify model establishment]	RNA from callus tissues, cells, and exosomes [NA], qPCR	Mouse (MC3T3‐E1 cells)
Zhang, J. (2021)^65^	miR‐187 ↑	Osteoporosis patients and healthy controls: blood samples [*n* = 33/group]	MSCs: osteogenic differentiation; transfection with miR‐187 mimics, miR‐187 inhibitor, BARX2 siRNA, NC	Mouse fracture model: ovariectomy and sham group; femoral fracture; injection of NC and miR‐187 lentivirus [*n* = 40]	X‐ray [NA; assess bone healing]; NA	RNA from serum samples, tissues [NA]; qPCR	Mouse (C57BL/6J); human
Hu, H. (2021)^90^	miR‐335 ↑		MC3T3 cells: NC‐treated, incubated with B‐EVs, BMMSCs transfected with miR‐335 inhibitor mock, BMMSCs transfected with miR‐335 inhibitor; MG63 cells: NC‐treated, incubated with B‐EVs, BMMSCs transfected with miR‐335 inhibitor mock, BMMSCs transfected with miR‐335 inhibitor	Mouse fracture model: femoral shaft fracture; WT + NC group, injection of EVs: WT + B‐EV, WT + EVs transfected with miR‐335 inhibitor‐NC, WT + EVs transfected with miR‐335 inhibitor, CD9–/– + NC, CD9–/– + B‐EV, CD9–/– + EVs transfected with miR‐335 inhibitor‐NC, CD9–/– + EVs transfected with miR‐335 inhibitor [NA]	X‐ray [0, 1, 2, 4 weeks after surgery; bone union of fracture site]; NA	RNA from tissues and cells [NA]; qPCR; microarray	Mouse (BMSCs, MC3T3 cells); human (MG63 cells)
Zhang, D. (2021)^79^	miR‐144‐5p ↓		BMDMs: collection of pelleted exosomes; BMSCs: BMDM‐derived exosomes uptake; BMSCs: osteogenic differentiation, transfection with siSMAD1, miR‐144‐5p mimic, miR‐144‐5p inhibitor or NC	Rat fracture model: T2DM group and normal group [*n* = 5/group]; transverse femur shaft fracture, injection of nBMDM‐Exos, dBMDM‐exos, dBMDM‐Exos + NC antagomir, dBMDM‐Exos + miR‐144‐5p‐antagomir at 1, 3, 5, 7 days after surgery [NA]	X‐ray [days 14, 21 after surgery; observe fracture region]; μCT [after removal of internal fixation at day 21; BV/TV to assess bone regeneration in the fracture site]	RNA from cells, exosomes, BMSCs and callus tissues [after 14 days of induction on BMSCs, callus tissues 21 days after surgery]; miRNA sequencing	Rat
Wang, X. (2021)^81^	miR‐214‐3p ↓		BMMSCs: exosome extraction; HUVECs: determine exosome uptake; BMMSCs and HUVECs: transfection with miR‐214‐3p mimic, miR‐214‐3p mimic‐NC, miR‐214‐3p inhibitor and inhibitor‐NC for 6 h	Mouse osteoporosis model: ovariectomy and sham‐operation; mechanical knee loading; BMSC isolation [NA]	NA; μCT [NA; evaluate bone microstructure, BV/TV, Tb.Th, Tb.N, Tb.Sp]	RNA from exosomes [NA]; exosomal miRNA sequencing; qPCR	Mouse
Zhang, Y. (2021)^123^	let‐7i‐5p ↑		BMSCs: transfection with siRNA220, siRNA738, siRNA1118, siRNA‐NC, let‐7i‐5p mimics, mimics NC, rno‐let‐7i‐5p inhibitor and inhibitor‐NC	Mouse fracture model: femur fracture, injection of agomiR‐let‐7i‐5p, agomiR‐NC or PBS [*n* = 60]	X‐ray [4 weeks after surgery; callus structure and fracture line]; NA	Total RNA from cells [NA]; qPCR	Mouse (C57BL/6, BMSCs)
Hu, L. (2021)^67^	miR‐92a‐3p ↑	Fracture patients: serum and callus samples; completely healed fracture, concomitant fracture and TBI, isolated fracture [*n* = 30 in total, 10/group]	Osteogenic precursor cells: transfection with agomiR‐92a‐3p, antagomiR‐92a‐3p, agomiR‐NC, antagomiR‐NC, siPI3K, si‐AKT, si‐IBSP	Mouse fracture model: femoral fracture; fracture group [*n* = 25] and fracture + TBI group [*n* = 5]; control group, injection of agomiR‐92a‐3p, antagomiR‐92a‐3 or PBS (negative control) on days 1, 3, 7	NA; μCT [NA; BV/TV, BMD, Tb.N, cortical area fraction, average cortical thickness, cortical bone area, Tb.Sp, Tb.Th, total cross‐sectional area]	Total RNA from cells and tissue samples [venous blood and callus samples on days 14 and 21 after surgery]; qPCR	Human; mouse (C57BL/6J, MC3T3‐E1 cells)
Ito, S. (2021)^100^	miR‐125b ↑			Mouse fracture model: development of miR‐125b in osteoblasts overexpressing TG mice; femoral shaft fracture in TG and WT mice [NA]	NA; μCT [NA; success of bone repositioning, BMD, BV/TV, Tb.Th, Tb.N, Tb.Sp]	RNA from blood	Mouse (C57BL/6J)
Wang, B. (2021)^68^	miR‐223‐3p ↑	Fracture patients: intra‐articular fracture [*n* = 42], hand fracture [*n* = 40], control group [*n* = 70]; blood samples collected at days 7, 14 and 21 after surgery	Osteoblasts: transfection with miR‐223‐3p mimic or inhibitor, miR‐NC, si‐FGFR2, si‐NC			Total RNA from cells and serum samples [days 7, 14 and 21 after surgery]; qPCR	Human; mouse (MC3T3‐E1 cells)
Yan, Z.‐W. (2021)^69^	miR‐182 ↓ (increased in tibial fractures)	Tibial plateau fracture patients [*n* = 80], healthy control [*n* = 80]; serum sample collection	Osteoblasts: transfection with miR‐182, anti‐miR‐182, miR‐NC or anti‐miR‐NC	Rat fracture model: fracture of the tibial plateau; control group, fracture group, injection of osteoblasts overexpressing miR‐182 or anti‐miR‐182 [*n* = 60 in total, *n* = 15/group]		Total RNA from serum samples and cells [NA]; qPCR	Human; rat (osteoblasts)
Wang, Y. (2021)^76^	miR‐467 ↓	Obese fracture patients [*n* = 20] and healthy controls [*n* = 20]; collect plasma samples	BMSC: control group (cultured in osteoinductive agents) and high‐fat group (high‐fat osteogenic induction), isolation of exosomes from BMSCs by collecting the supernatant; BMSCs: osteogenic differentiation	Obese fracture mouse model: model (high‐fat diet) and control group (normal diet); tibial fracture; injection of HFD‐Exos or PBS near to the fracture [*n* = 8/group]		Total RNA from callus tissues [NA]; qPCR	Human; mouse (C57BL/6J)
Zhang, Y. (2021)^136^	miR‐331‐3p ↑		MOBs: transfection with miR‐331‐3p mimic and inhibitor	Rabbit fracture model: 2 mm hole in the tibia and injection of *Staphylococcus aureus* into this hole to induce infection; tibia samples collected 28 days after infection	NA; μCT [28 days after infection; BV/TV, Tb.Th]	Total RNA from tibia tissue samples and cells [28 days after infection]; qPCR	Rabbit; mouse (mouse calvaria osteoblasts)
Yang, W. (2021)^75^	miR‐100‐5p ↓	Patients with nontraumatic osteonecrosis of the femoral head and femoral neck fracture patients [*n* = 40/group]: tissue samples, extraction of exosomes from bone tissues	hBMSCs: culturing with exosomes isolated from patient bone tissue; transfection with NC, agomiR‐100‐5p, antagomiR‐100‐5p, siBMPR2, WT BMPR2 and mutant type BMPR2 plasmids; osteogenic differentiation, adipocyte differentiation induction after the cells reached 100% confluency; HUVECs: cultured with exosomes and study of the tube formation	Rat model: injection of exosomes from patient tissue, or PBS as control; femoral head harvested [*n* = 30]	NA; μCT [after 8 weeks of treatment; BV/TV, Tb.Sp, Tb.Th, Tb.N]	Total RNA [NA]; qPCR; miRNA sequencing	Human (BMSCs, HUVECs); rat
Huang, Y. (2021)^112^	miR‐206 ↑		BMSCs: transfection with miR‐206 mimic, miR‐206 inhibitor and their NC; isolation of exosomes from BMSCs; osteoblasts: isolated from knee joint samples of the mice, co‐culture with BMSC‐Exos (non‐transfected BMSC‐Exos, mimic‐NC‐Exos (miR‐206 mimic, mimic‐NC), miR‐206 mimic, sh‐NC, sh‐Elf3 group	Mouse osteoarthritis model: collagen‐induced arthritis; injection of exosomes in articular cavity of the knee; sham group, treatment groups with cut off the anterior ligament of the knee exosomal injection with non‐transfected BMSC‐Exos, NC‐Exos, transfected BMSC‐Exos (miR‐206 mimic and inhibitor) [NA]	NA; μCT [NA; BMD, BV/TV, Tb.Sp, Tb.Th, Tb.N]	Total RNA from bone and blood samples [8 weeks after modelling and treatment of the mice]; qPCR	Mouse (C57BL/6: BMSCs)
Dai, Z.Q. (2022)^164^	miR‐100 ↓		BMSCs from sham group or OVX group: transfection with miR‐100 inhibitor with or without AKT inhibitor for 48 h	Osteoporosis mouse model: ovariectomy or sham operation; tibia taken from mice [NA]	NA; μCT [after surgery; confirm osteoporosis model by BV/TV of distal femur]	Total RNA from tissues and cells [48 h after transfection]; qPCR	Mouse (C57BL/6J)

*Note*: ↓↑ denotes promoting/decreasing bone healing. NA denotes data not available.

Abbreviations: AKT, RAC‐alpha serine/threonine‐protein kinase; AMO, anti‐microRNA antisense oligodeoxyribonucleotide; ASCs, adipose‐derived stem cells; ASO, antisense oligonucleotide; BMD, bone mineral density; BMDM, bone marrow‐derived macrophages; BMM, bone marrow‐derived macrophages; BMMSC/BMSCs, bone mesenchymal stem cells; BN, bone non‐union; BV, bone volume; DM, diabetes mellitus; DMEM, Dulbecco's modified Eagle medium; EPC, endothelial progenitor cells; EV, extracellular vesicles; Exos, exosomes; FBS, foetal bovine serum; FGF, fibroblast growth factor; HucMSCs, human umbilical cord MSCs; HUVEC, human umbilical vein endothelial cell; IBSP, integrin binding sialoprotein; IGF, insulin‐like growth factor; IL, interleukin; MOB, murine osteoblasts; MSC, mesenchymal stromal cell; NC, negative control; OVX, ovariectomised; PBS, phosphate buffered saline; PI3K, phosphoinositid 3‐kinase/serine‐threonine‐kinase; PTEN, phosphatase and tensin homologue deleted on chromosome 10; TBI, traumatic brain injury; Tb.N, trabecular number; Tb.Sp, trabecular separation; Tb.Th, trabecular thickness; TG, transgenic; TV, total volume; T2DM, type 2 DM; WT, wild‐type; μCT, micro‐computed tomography.

For ‘microRNA and fracture non‐union’, records identified were *n* = 17 on PubMed, *n* = 9 on Web of Science, *n* = 7 on EBSCO and *n* = 63 on Scopus, and 13 full texts fulfilled the inclusion criteria (Table 2). Additionally, six results from the keyword search ‘microRNA and fracture healing’ were included in the table of non‐union fractures, as they focused on non‐unions. In summary, as depicted in Table 2, only 19 full‐text articles were found focusing specifically on miRNAs in fracture delay or non‐union.

**TABLE 2:** Differentially expressed microRNAs (miRNAs) in non‐union fractures

Author (year)	miRNA (miRNA number; ↓↑ in non‐union)	Clinical screening (patient groups [number of patients])	In vitro experiment groups (cell type: groups during experiment)	Animal model (type of model; treatment on the model [number of replicates])	For animal model: healing follow‐up (X‐ray [timepoint; analysis on X‐ray]; μCT [timepoint; measurements on μCT])	miRNA analysis (RNA isolation from cells/tissue [timepoint of isolation]; qPCR; microarray analysis)	Cell origin (cell line; cell type)
Waki, T. (2015)^140^	miR‐31a‐3p (↑), ‐146a‐5p (↑), ‐146b‐5p (↑), ‐223‐3p (↑)			Rat fracture model: femoral fracture, non‐union group with cauterised periosteum (2 mm distance) for 28 days; tissue samples collected on days 3, 7, 14, 21 and 28 after fracture [*n* = 94]	X‐ray [days 0 and 28 after surgery]; NA	Total RNA [days 3, 7, 14, 21 and 28 after fracture]; qPCR; microarray	Rat
Waki, T. (2016)^129^	miR‐181d‐5p (↓), ‐181a‐5p (↓), ‐140‐5p (↓), ‐451a (↓), ‐208b‐3p (↓), ‐743b‐5p (↓), ‐879‐3p (↓), ‐140‐3p (↓)			Rat femoral fracture model [*n* = 12]; shaft fracture, unhealing fracture group (cauterisation of the periosteum) [*n* = 5/group and timepoint]	X‐ray [days 0 and 28 after surgery]	Total RNA from the fracture site [days 3, 7, 10, 14, 21 and 28 after fracture]; microarray analysis [day 14]; qPCR	Rat
Chen, H. (2017)^134^	miR‐628‐3p (↑), ‐149 (↑), ‐221 (↑), ‐654‐5p (↑); let‐7b (↑), ‐220b (↑), ‐513a‐3p (↑), ‐551a (↑), ‐576‐5p (↑), ‐1236 (↑), kshv‐miR‐K12‐6‐5p (↑) (in non‐union)	Patients with atrophic non‐union [*n* = 3], normal fracture healing [*n* = 3]; samples from scar tissue	Osteoblasts: transfection with miR‐628‐3p miRNA‐mimic or miR‐654‐5p mimics or miR‐NC			Total RNA from cells and tissues [NA]; qPCR	Human (MG63 cells)
Peng, H. (2018)^133^	miR‐133a ↓	Fracture non‐union patients [*n* = 40], fracture healing group [*n* = 40]; removing internal fixator in control group	Osteoblasts: transfection with anti‐BMP2 and anti‐RUNX2 antibody			Total RNA from bone tissue [NA]; qPCR	Human; mouse (MC3T3‐E1 cells)
Guo, P.‐Y. (2019)^142^	miR‐140‐5p ↑		ASCs: osteogenic differentiation, transduction with lentiviral plasmids with NC or miR‐140‐5p	Rat atrophic non‐union model: femoral fracture with destroyed periosteum (cauterisation); atrophic group, NC group, injection of ASC with NC or with miR‐140‐5p‐TuD for 4 weeks [*n* = 36 in total]	NA; μCT [NA; BV/TV, Tb.Th, Tb.Sp, Tb.N]	Total RNA from cells [NA]; qPCR	Rat; human (ASCs)
Sun, L. (2019)^143^	miR‐26a ↑		BMMSCs: incubate with CD44/CD90/CD31/CD34	Rat non‐union model: femur fracture, control and non‐union group with removing of the femur periosteum; injection of miR‐26a or NC in non‐union rats into the surrounding area of the fracture site for 8 weeks [*n* = 6]	X‐ray [2, 4 and 8 weeks after surgery; NA]; NA	Total RNA from bone tissues and cells [8 weeks after surgery]; qPCR	Rat (BMMSCs)
Long, H. (2019)^138^	miR‐381 ↓	Atrophic non‐union patients [*n* = 10] and standard healing fracture patients [*n* = 10]	BMSCs: transfection with miR‐281‐3p mimics or miR‐381‐3p inhibitor	Rat fracture model: mid‐diaphysis femoral fracture, injection of miR‐381 antagomir or NC antagomir into the fracture site on days 4, 7, 11 and 14 [NA]	X‐ray [days 7 and 14 after fracture; fracture line, callus formation]; NA	Total RNA from cells and tissues [day 14 after fracture]; qPCR; microarray [*n* = 3/tissue sample group]	Human (BMSCs); rat
Orth, M. (2019)^141^	44 miRNAs: relevant for non‐union formation			Mouse fracture model: femoral fracture; union group with gap of 0.25 mm [*n* = 7] and non‐union group with gap size of 1.8 mm [*n* = 7]	X‐ray [day 7; exclude dislocation of metallic implants]; NA	Total RNA from cells and tissues [7 days after surgery]; qPCR; microarray	Mouse
Takahara, S. (2020)^139^	miR‐221‐3p (↑), ‐339‐3p (↑), ‐376a‐3p (↑), ‐379‐5p (↑), ‐451‐5p (↑)			Rat fracture model: DM group and control group (sham treatment) [*n* = 54/group]; closed femoral shaft fracture; sacrificed on days 7, 14, 21 and 28 after fracture [*n* = 8/group and timepoint]	X‐ray [days 7, 14, 21 and 28 after fracture; evaluation of callus of the four cortices]; NA	Total miRNA from callus and cells [days 5, 7, 11, 14, 21 and 28 after fracture]; qPCR; microarray [days 5 and 11 after fracture]	Rat
Xiong, Y. (2020)^145^	miR‐6979‐5p ↑		Osteoblasts: transfection with agomiR‐6979‐5p, agomiR‐NC, antagomiR‐6979‐5p, antagomiR‐NC, lncRNA Rhno1, silncRNA Rhno1 and siRNA‐NC	Mouse fracture model: mid‐diaphysis femoral fracture; half euthanised after 14 days after surgery, the other half after 21 days; injection of Cy3‐labelled agomiR‐6979‐5p on days 0, 4, 7, 10 and 14 [NA]	X‐ray [days 0, 4, 7, 10 and 14 after surgery]; μCT [NA; BV/TV, BMD]; NA	Total RNA from cells and tissue [NA]; qPCR	Mouse (C57BL/6J, MC3T3‐E1 cells)
Xie, H. (2020)^132^	miR‐1323 ↑ (in non‐union)	Atrophic non‐union fracture specimens [*n* = 5], standard healing fracture specimens [*n* = 5] collected during open reduction or internal fixation	MSCs: osteogenic differentiation for 7 days; transfection with miR‐1323‐3p inhibitor, miR‐1323‐3p mimics or their NC for 7 days; lentiviral infection with BMP4, SMAD4 or NC for 7 days	Rat fracture model: mid‐diaphyseal femur fracture; injection of NC antagomiR or miR‐1323 antagomir around the fracture site on days 4, 7 and 11 after surgery [NA]	X‐ray [days 7 and 14 after surgery; callus formation, fracture gap bridging/fracture line]; NA	Total RNA from cells and callus tissue [7 days after osteoinductive culturing; day 14 after surgery]; qPCR	Human (MSCs); rat
Xiong, Y. (2020)^131^	miR‐193a‐3p ↑ (in non‐union)	Bone union patients; BN patients, healthy patients [*n* = 6/group]; blood sample collection on days 1 and 3 after surgery	MSCs: transfection with siRNA MAPK10, agomiR‐193a‐3p or antagomiR‐193a‐3p	Mouse fracture model: mid‐diaphysis femoral fracture, sacrificed on days 14 and 21 after surgery; injection of PBS (control group), agomiR‐193a‐5p, antagomiR‐193a‐3p on days 1, 3 and 7 after surgery [*n* = 20 in total]	NA; μCT [NA; BV/TV; BMD]	Total RNA from cells and tissue samples [NA]; qPCR	Mouse (C57BL/6J); human (MSCs)
Ouyang, Z. (2020)^135^	miR‐205‐5p ↓	Patients with non‐union [*n* = 10] and normal fracture healing [*n* = 14]	BMSCs: lentiviral‐mediated overexpression of pLVX‐IRES‐Puro and pLKO.1‐Vektor; HUVECs: adding of conditional medium and investigating tube formation	Mouse fracture model: femoral monocortical defect, transplantations of BMSCs into osseous hole for 1 month [NA]	NA; μCT [1 month after fracture; BV/TV; BMD]	Total RNA from tissue samples and cells [NA]; qPCR; RNA sequencing (on the tissue samples)	Human (BMSCs, HUVECs); mouse
Li, G.J. (2020)^144^	miR‐149 ↑		BMMSCs: osteogenic differentiation, transfection with mimic‐NC, miR‐149 mimic, inhibitor‐NC, miR‐149 inhibitor, sh‐NC and sh‐H19 for 48 h	Rat fracture model: bone defect on femoral shaft, filling the defect with polymethyl methacrylate cement (membrane‐induced osteogenic differentiation) or untreated (sham group) [NA]		Total RNA from cells [48 h after transfection]; qPCR	Rat
Wei, J.Q. (2020)^53^	miR‐149 (↑), ‐221 (↑), ‐628‐3p (↑), ‐654‐5p (↑) (in non‐union); hsa‐let‐7b (↓), ‐220b (↓), ‐513a‐3p (↓), ‐551a (↓), ‐576‐5p (↓), ‐1236 (↓), ‐K12‐6‐5p (↓) (in non‐union)	Patients with non‐union [*n* = 3], fracture healed patients [*n* = 3]: non‐union or callus tissues collected	BMSCs: transfection with miR‐149, ‐221, ‐628‐3p, ‐654‐5p or their NC for 48 h			Total RNA from tissue samples [NA]; qPCR; microarray	Human (BMSCs)
Dai, Y. (2021)^125^	miR‐649 (↑), ‐29b‐3p (↑), ‐498 (↑), ‐365a‐5p (↑), ‐328‐5p (↑), ‐345‐3p (↑)	Patients with infected non‐union of the tibia, healthy control with closed tibial fracture [NA]				Total RNA from tissue samples and cells [NA]; qPCR; microarray	Human
Chen, J. (2021)^130^	miR‐214 ↓	Tibia plateau fracture; unhealed fractures within 4 months [*n* = 42]	Osteoblasts: transfection with sh‐lncRNA HAGLR, sh‐HAGLR‐NC, miR‐214‐3p mimic and mimic‐NC	Mouse fracture model: tibial fracture; sham group [*n* = 5], tibial fracture and injection of pc‐HAGLR after surgery [*n* = 60 in total]		Total RNA [48 h after transfection]; qPCR	Mouse (C57BL/6, MC3T3‐E1 cells)
Zhang, Y. (2021)^137^	miR‐212 ↓ (in non‐union)	Patients with delayed fracture healing in femoral neck fracture [*n* = 30]: serum samples collected	Osteoblastic osteosarcoma cells: transfection with siRNA against MALAT1, si‐NC, miR‐212 mimics, miR‐212 inhibitor, si‐SOX6 and NC			Total RNA from serum samples and cells [NA]; qPCR; microarray (lncRNAs)	Human (MG‐63 cells)
Zhang, Y. (2022)^136^	miR‐135 ↓	Unhealed fracture patients (about 6 months): callus samples [*n* = 4]	MC3T3‐E1: transfection with shXIST, transfection or infection with miR‐135 inhibitor or CREB1 lentiviral overexpression vectors	Mouse fracture model: tibial fracture; sham group (without treatment), model, NC and shXIST group [*n* = 80 in total; 20/group]	X‐ray [7, 14 and 21 days after fracture; NA]; μCT [7, 14 and 21 days after surgery; BV/TV; BMD]	Total RNA on callus samples and cells [*n* = 7, 14, 21 after fracture]; qPCR; microarray	Human; mouse (C57BL/6J, MC3T3‐E1 cells)

*Note*: ↓↑ denotes promoting/decreasing bone healing. NA denotes data not available.

Abbreviations: ASCs, adipose‐derived stem cells; BMD, bone mineral density; BMP, bone morphogenic protein; BMSCs, bone mesenchymal stem cells; BV, bone volume; DM, diabetes mellitus; HUVEC, human umbilical vein endothelial cell; lncRNA, long noncoding RNA; MSC, mesenchymal stromal cell; qPCR, quantitative polymerase chain reaction; RUNX2, runt‐related transcription factor 2; SMAD, mothers against decapentaplegic homologue; TV, total volume, Tb.N, trabecular number, Tb.Th, trabecular thickness, Tb.Sp, distance between trabeculae, μCT: micro‐computed tomography.

### 3.2 miRNAs in fracture healing: clinical screening

Twenty‐four studies analysed miRNAs in blood or tissue samples (bone biopsies) of fracture patients.^45^, ^54^, ^55^, ^56^, ^57^, ^58^, ^59^, ^60^, ^61^, ^62^, ^63^, ^64^, ^65^, ^66^, ^67^, ^68^, ^69^, ^70^, ^71^, ^72^, ^73^, ^74^, ^75^, ^76^ Blood or tissue samples were collected during surgery, subsequently RNA was extracted, and validation of miRNAs was performed. In detail, *n* = 18 studies^45^, ^55^, ^56^, ^57^, ^59^, ^60^, ^61^, ^62^, ^63^, ^65^, ^66^, ^67^, ^68^, ^69^, ^70^, ^71^, ^74^, ^76^ analysed blood samples and *n* = 11 studies^45^, ^54^, ^55^, ^64^, ^65^, ^66^, ^67^, ^72^, ^73^, ^75^, ^76^ analysed samples collected from bone biopsies. Five studies^45^, ^54^, ^55^, ^65^, ^67^ analysed both blood and tissue samples. Twenty studies^54^, ^55^, ^56^, ^57^, ^58^, ^59^, ^60^, ^61^, ^62^, ^63^, ^64^, ^65^, ^66^, ^67^, ^68^, ^69^, ^73^, ^74^, ^75^, ^76^ validated their findings from clinical screening in an in vitro model, eight studies^54^, ^64^, ^65^, ^67^, ^69^, ^73^, ^75^, ^76^ used an animal model for validation, and all of the studies investigating an animal model for validation also performed additional in vitro validation.

**TABLE 3:** Differentially expressed microRNAs (miRNAs) in fracture healing (A) and non‐union (B)

Validated miRNA	Independent reports (*n*)	Author (year), ↑/↓
**(A) Fracture healing**		
**Upregulated**		
miR‐125b(‐3p/5p)	4	Furuta, T. (2016)^91^ ↑, Bourgery, M. (2021)^78^ ↑, Seeliger, C. (2014)^45^ ↑, Ito, S. (2021)^100^ ↑
miR‐148(a‐3p)	3	Liu, H. (2018)^84^ ↑, Seeliger, C. (2014)^45^ ↑, Bourgery, M. (2021)^78^ ↑
miR‐25(‐3p)	3	Jiang, Y. (2020)^96^ ↑, Lang, Y. (2019)^95^ ↑, Seeliger, C. (2014)^45^ ↑
miR‐100(‐5p)	3	Seeliger, C. (2014)^45^ ↑, Dai, Z.Q. (2022)^164^ ↑, Yang, W. (2021)^75^ ↑
miR‐187(‐3p)	2	Zhang, J. (2021)^65^ ↑, Yuan, H.F. (2015)^72^ ↑
miR‐206	2	He, B. (2016)^82^ ↑, Huang, Y. (2021)^112^ ↑
miR‐19b	2	Zarecki, P. (2020)^71^ ↑, Huang, Y. (2021)^99^ ↑
miR‐23(a‐3p)	2	Seeliger, C. (2014)^45^ ↑, Zarecki, P. (2020)^71^ ↑
miR‐24(‐3p)	2	He, B. (2016)^82^ ↑, Seeliger, C. (2014)^45^ ↑
miR‐26a(‐5p)	2	Li, Y. (2015)^102^ ↑, Xiong, Y. (2019)^73^ ↑
miR‐34a(‐5p)	2	Yuan, H.F. (2015)^72^ ↑, Liu, H. (2019)^118^ ↑
miR‐122(a‐5p)	2	Seeliger, C. (2014)^45^ ↑, Silva, A.M. (2018)^86^ ↑
miR‐136(‐3p/5p)	2	Bourgery, M. (2021)^78^ ↑, Yu, H. (2021)^122^ ↑
**Downregulated**		
miR‐124(a‐3p)	3	Cui, Y. (2019)^108^ ↓, Zou, L. (2017)^57^ ↓, Seeliger, C. (2014)^45^ ↓
miR‐144(‐3p/5p)	3	Bourgery, M. (2021)^78^ ↓, He, B. (2016)^82^ ↓, Zhang, D. (2021)^79^ ↓
miR‐494(‐3p)	2	He, B. (2016)^82^ ↓, Hadjiargyrou, M. (2016)^103^ ↓
miR‐497(‐3p)	2	Ji, X. (2021)^62^ ↓, He, B. (2016)^82^ ↓
**Up and downregulated**		
miR‐21(‐5p)	9	Liu, Y. (2019)^109^ ↑, Sheng, J. (2019)^115^ ↓, Sun, Y. (2015)^101^ ↑, Hadjiargyrou M. (2015)^103^ ↑, Sun, X. (2020)^46^ ↑, Seeliger, C. (2014)^45^ ↑, Zarecki, P. (2020)^71^ ↑, Strauss, F.J. (2020)^128^ ↓, Silva, A.M. (2018)^86^ ↑
miR‐214(‐3p/5p)	8	Xin, Z. (2020)^55^ ↓, Zhou, L.‐G. (2019)^111^ ↓, Teng, J.‐W. (2018)^113^ ↓, Li, Q.S. (2017)^63^ ↑, Li, K.‐C. (2016)^104^ ↓, Chen, J. (2021)^130^ ↓, Wang, X. (2021)^81^ ↓, Li, K.C. (2017)^106^ ↓
miR‐140(‐3p/5p)	7	Waki, T. (2016)^129^ ↓, Jiao, J. (2020)^120^ ↑, Liu, Q.‐P. (2019)^48^ ↑, Bourgery, M. (2021)^78^ ↓, Hadjiargyrou, M. (2016)^103^ ↑, Li, K.‐C. (2016)^104^ ↓, Takahara, S. (2018)^85^ ↓
miR‐223(‐3p/5p)	5	Bourgery, M. (2021)^78^ ↓, He, B. (2016)^82^ ↓, Seeliger, C. (2014)^45^ ↑, Wang, B. (2021)^68^ ↑, Dietz, C. (2019)^70^ ↑
miR‐142(a‐3p/5p)	4	Bourgery, M. (2021)^78^ ↓, He, B. (2016)^82^ ↓, Hadjiargyrou, M. (2016)^103^ ↓, Tu, M. (2017)^93^ ↑
miR‐181a(‐3p/5p)	4	Waki, T. (2016)^129^ ↓, Takahara, S. (2018)^85^ ↑, Yuan, H.F. (2015)^72^ ↑, Bourgery, M. (2021)^78^ ↑
miR‐335(‐5p)	3	Janko, M. (2019)^166^ ↓, Hu, H. (2021)^90^ ↑, Zarecki, P. (2020)^71^ ↑
miR‐92a(‐3p)	3	Murata, K. (2014)^64^ ↑, Janko, M. (2019)^166^ ↑, Hu, L. (2021)^67^ ↓
miR‐22(‐3p)	3	He, B. (2016)^82^ ↓, Zhang, X. (2020)^47^ ↑, Weilner, S. (2015)^66^ ↑
miR‐532‐3p	2	Zarecki, P. (2020)^71^ ↑, Silva, A.M. (2018)^86^ ↓
miR‐222(‐3p)	2	Jiang, C. (2020)^97^ ↓, Takahara, S. (2018)^85^ ↑
miR‐328‐3p	2	Xie, W. (2020)^56^ ↓, Weilner, S. (2015)^66^ ↑
miR‐429	2	Huang, J. (2016)^105^ ↑, Silva, A.M. (2018)^86^ ↓
miR‐181c(‐3p/5p)	2	Bourgery, M. (2021)^78^ ↓, Yuan, H.F. (2015)^72^ ↑
miR‐182(‐5p)	2	Bourgery, M. (2021)^78^ ↑, Yan, Z.‐W. (2021)^69^ ↓
miR‐10a‐5p	2	Bourgery, M. (2021)^78^ ↓, Weilner, S. (2015)^66^ ↑
**(B) Non‐union**		
**Upregulated**		
miR‐149	2	Li, G.J. (2020)^144^ ↑, Chen, H. (2017)^134^ ↑
miR‐221‐3p	2	Takahara, S. (2020)^139^ ↑, Chen, H. (2017)^134^ ↑
miR‐31‐5p	2	Waki, T. (2015)^140^ ↑, Orth, M. (2019)^141^ ↑
**Downregulated**		
miR‐451‐5p	2	Waki, T. (2015)^140^ ↓, Takahara, S. (2020)^139^ ↓
**Up and downregulated**		
miR‐140‐5p	3	Guo, P.‐Y. (2019)^142^ ↑, Waki, T. (2016)^129^ ↓, Orth, M. (2019)^141^ ↑

**FIGURE 2:**
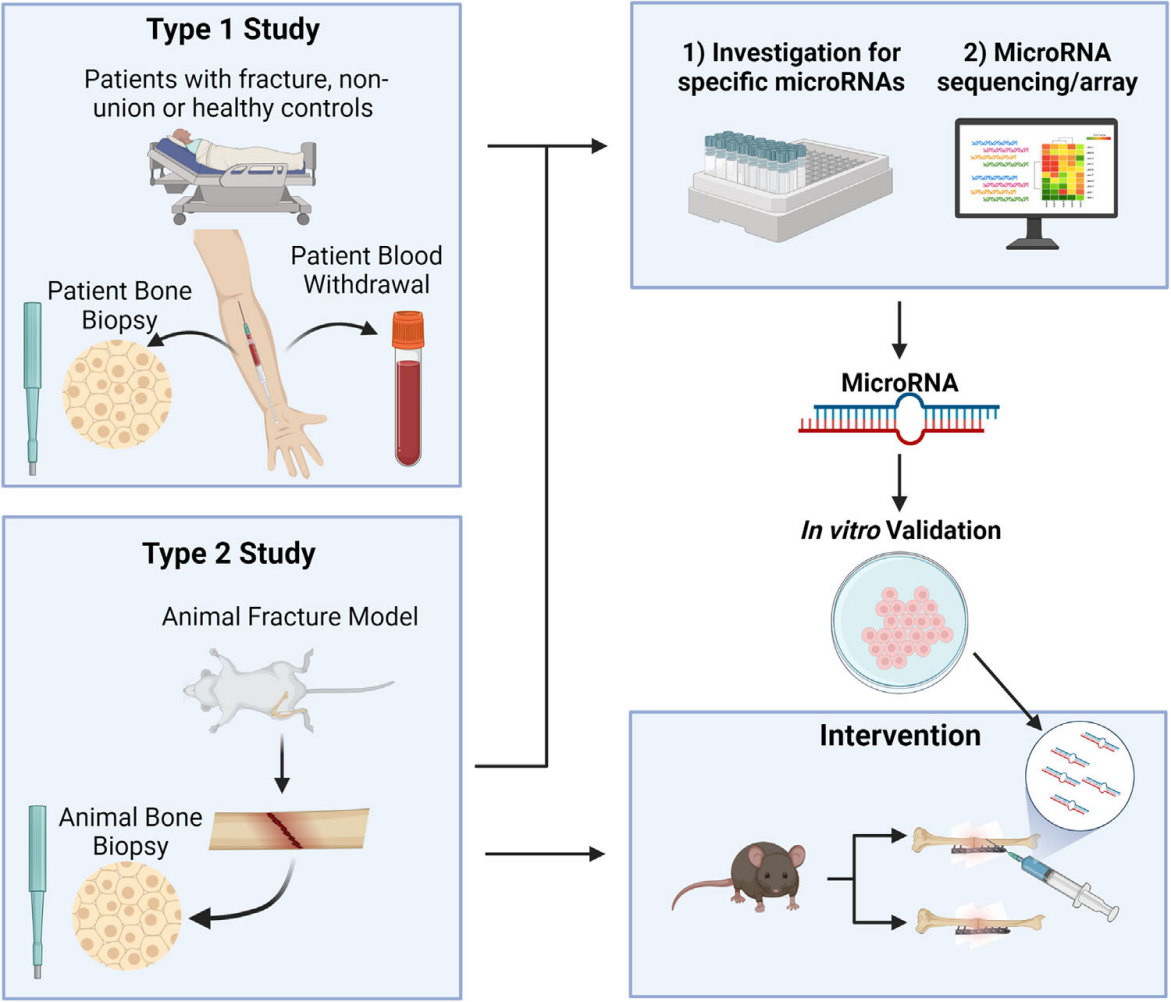
Different study types to investigate microRNAs (miRNAs) during fracture healing process. Type 1 studies focused on patient screenings using bone biopsy or blood sample analysis. Type 2 studies used animal fracture models and analysed callus tissues. Analyses were either performed by miRNA sequencing or microarray, followed by in vitro validation of the miRNA and an animal study. Some studies performed an interventional animal study by injecting miRNAs into the animal fracture site for validation.

Since the different studies provide different opportunities to find possible miRNAs involved in fracture healing, Figure 2 gives a complete overview about the investigations carried out and the different study types.

### 3.3 miRNAs in fracture healing: sequencing and microarray analysis

Six out of 82 studies performed RNA sequencing from tissue or cell samples to identify differentially regulated miRNAs during fracture healing.^75^, ^77^, ^78^, ^79^, ^80^, ^81^ One study included both blood and tissue samples from patients,^75^ while five studies^77^, ^78^, ^79^, ^80^, ^81^ performed the analysis in animal models. Five of those six studies^75^, ^77^, ^79^, ^80^, ^81^ validated their findings in a cellular model and by performing qPCR analysis. Thirteen studies performed microarray analysis instead of sequencing.^45^, ^49^, ^72^, ^77^, ^82^, ^83^, ^84^, ^85^, ^86^, ^87^, ^88^, ^89^, ^90^ For example, Seeliger et al.^45^ performed microarray analysis on blood serum samples of fracture patients or osteoporotic fracture patients and screened for 83 different miRNAs. Takahara et al.^85^ performed microarray analysis on callus samples collected from diabetic fractures in rats. In total, two studies performed microarray screening in blood or tissue samples.^45^, ^72^ Eleven studies included animal models for microarray analysis,^49^, ^77^, ^82^, ^83^, ^84^, ^85^, ^86^, ^87^, ^88^, ^89^, ^90^ while 12 studies validated their findings in a cell model and by qPCR.^49^, ^72^, ^77^, ^82^, ^83^, ^84^, ^85^, ^86^, ^87^, ^88^, ^89^, ^90^

### 3.4 miRNAs in fracture healing: fracture models

Sixty‐six out of 82 studies validated their findings by performing an animal fracture model. The in vivo models consisted of a surgery to implement a fracture and a subsequent follow‐up analysis to monitor the fracture healing. Among the identified studies, femur fracture in mid‐diaphysis/shaft being created by osteotomy was the most frequent approach (*n* = 22^27^, ^64^, ^73^, ^79^, ^80^, ^82^, ^84^, ^85^, ^87^, ^88^, ^89^, ^90^, ^91^, ^92^, ^93^, ^94^, ^95^, ^96^, ^97^, ^98^, ^99^, ^100^), while 16 studies^54^, ^83^, ^85^, ^86^, ^101^, ^102^, ^103^, ^104^, ^105^, ^106^, ^107^, ^108^, ^109^, ^110^, ^111^, ^112^ did not specify the exact location of the fracture.

Thirty‐nine of the studies that included an animal fracture model examined the effect of selected miRNAs directly on fracture healing by injection of miRNAs or their inhibitors into the fracture site.^27^, ^46^, ^47^, ^48^, ^54^, ^65^, ^69^, ^73^, ^77^, ^79^, ^83^, ^84^, ^88^, ^89^, ^90^, ^92^, ^93^, ^94^, ^95^, ^98^, ^99^, ^101^, ^102^, ^105^, ^109^, ^110^, ^111^, ^112^, ^113^, ^114^, ^115^, ^116^, ^117^, ^118^, ^119^, ^120^, ^121^, ^122^, ^123^ Seven studies used exosomes to deliver the identified miRNAs to the fracture site.^49^, ^76^, ^77^, ^79^, ^91^, ^97^, ^99^

Osteoporosis patients with a lower BMD presented low‐impact fractures, such as spine fractures or femoral neck fractures.^124^ Nine studies^46^, ^47^, ^65^, ^81^, ^102^, ^104^, ^106^, ^111^, ^125^ used an osteoporosis fracture model to screen and validate differentially expressed miRNAs in vivo. This was done by ovariectomy in mice or rats, causing a loss in BMD to study miRNAs being differentially expressed in fractures under osteoporotic conditions. The osteoporosis model was either implemented to isolate bone marrow‐derived MSCs (BMSCs) afterwards and perform transfection with miRNAs in vitro,^81^, ^104^ or the model was directly used to inject miRNAs or their inhibitors to study the fracture outcome in osteoporosis‐induced animals.^65^, ^111^

To assess the fracture healing process in animal models, X‐ray and micro‐computed tomography (μCT) are commonly used, with the majority of studies combining both methods. X‐ray was usually performed to control the model immediately after the first surgery where the fracture was implemented. Subsequently, X‐ray was used during the follow‐up to monitor the fracture healing, analysing callus formation, bridging of the fracture gap and callus volume.^64^ There are only a few common timepoints during the experiment to perform an analysis based on μCT and usually the animals are euthanised for this purpose. Recently, in vivo μCT protocols have been developed that allow for longitudinal monitoring of healing progression.^126^ This could be worth to be included in further studies investigating the fracture healing process. Common μCT analysis includes bone volume (BV), tissue volume (TV), BV/TV and BMD.^121^

In terms of mouse fracture models, C57BL/6N was the most common mouse strain (*n* = 20). Twenty studies performed a fracture model in rats, without clearly specifying the animal strain, and four studies implemented an osteoporotic fracture model.^46^, ^104^, ^106^, ^111^ Two studies performed a fracture model in rabbits.^115^, ^127^

### 3.5 miRNAs in fracture healing: in vitro experiments

To validate miRNAs involved in differentiation processes, 65 studies included in vitro experiments. Fifty‐seven studies performed transfection of BMSCs or osteoblasts with the miRNAs of interest, with lipofection as a common method to deliver miRNA agonists or antagonists.^54^ A large heterogeneity in cell origins and cell types used was detected. Thirty‐three studies used primary bone marrow mesenchymal stem cells isolated from human subjects, mice or rats. Only five studies worked with human primary cells to validate the effect of miRNAs on the differentiation process, which is probably the most clinically translatable in vitro experiment.^47^, ^66^, ^87^, ^91^, ^99^ Most studies (*n* = 43) validated their findings using cell lines. Two studies performed experiments with human embryonic kidney cells HEK293,^84^, ^121^ one used the osteosarcoma cell line MG‐63^90^ and one study used human umbilical vein endothelial cells (HUVECs).^87^ Studies on mouse cells used either BMSCs (*n* = 13) or the precursor osteoblast cell line MC3T3‐E1 (*n* = 18). One study performed experiments on mouse embryonic C3H10T1/2 cells.^120^

### 3.6 Summary of validated miRNAs in fracture healing

**FIGURE 3:**
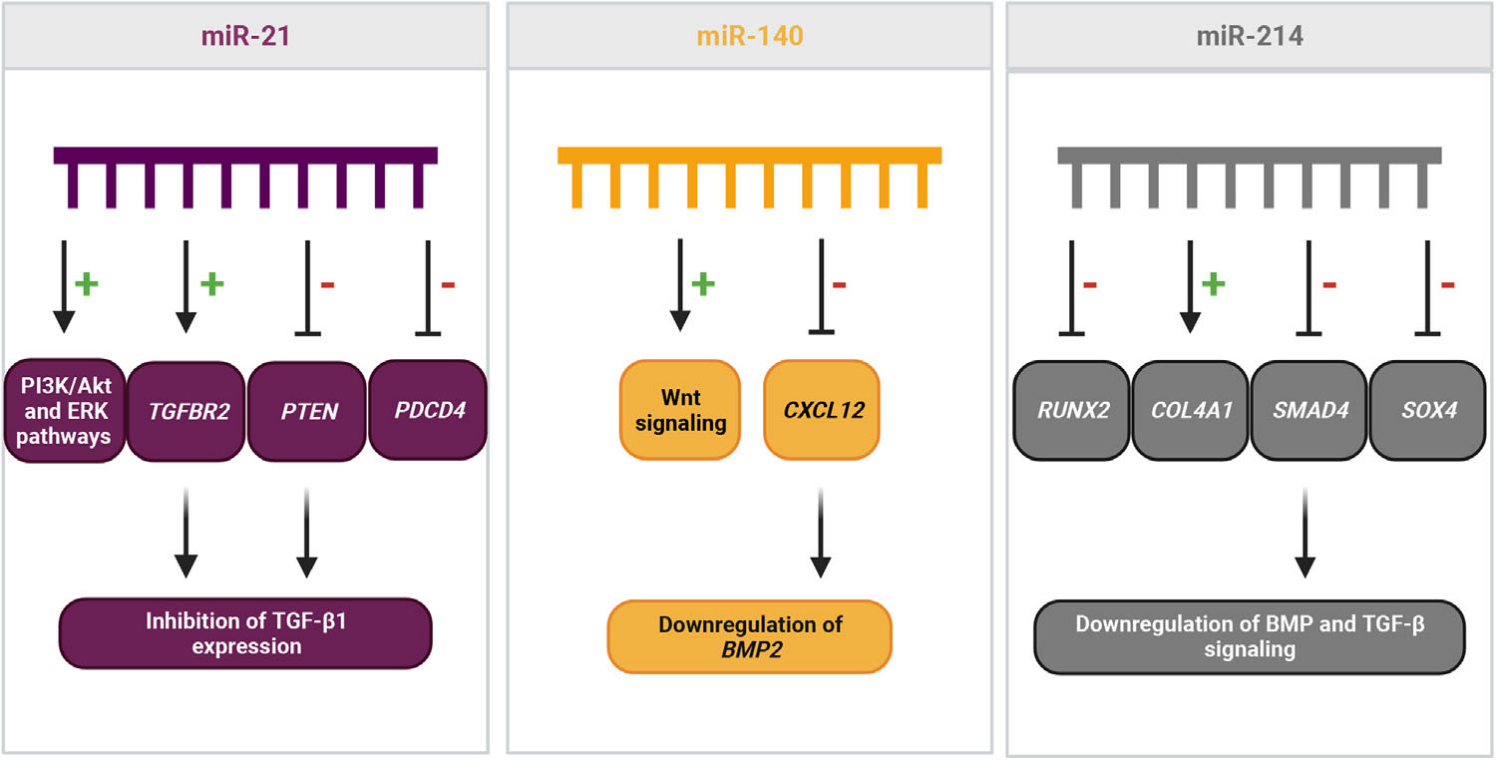
MicroRNAs (miRNAs) in fracture healing. Mechanism of action of miR‐21, miR‐140 and miR‐214 that are involved in fracture healing. ‘+’ indicates promotion, while ‘–’ indicates inhibition of the target gene or pathway. This regulation does not necessarily represent the direct target of the miRNA but rather the overall downstream pathway regulation. miR‐21 regulates the phosphoinositid‐3kinase/serine‐threonine‐kinase (PI3K) and extracellular signal‐related kinase (ERK) pathways, transforming growth factor‐β‐receptor 2 (*TGFBR2*), phosphatase and tensin homologue deleted on chromosome 10 (*PTEN*) and programmed cell death 4 (*PDCD4*) expression. This leads to inhibition of TGF‐β1 expression. miR‐140 regulated genes lead to an upregulation of bone morphogenic protein 2 (*BMP2*) and inhibition of stromal cell‐derived factor 1 (SDF‐1/*CXCL12*) followed by an upregulation of *BMP2*. miR‐214 combines the effects of both miR‐21 and miR‐140 as it downregulates the BMP and TGF‐β signalling.

Table 1 lists all the miRNAs described as differentially expressed during fracture healing processes. The miRNAs that are more often found to be regulated during bone remodelling in screenings and sequencing should be further validated and examined to find possible biomarkers predicting the outcome of the fracture healing process. Table 3 presents an overview of the most described miRNAs that are differentially expressed in fracture healing (A) and non‐union fractures (B). Overall, 121 different miRNAs were identified in 82 different studies as differentially expressed in fracture healing. Eighteen miRNAs were identified in at least two independent studies, eight were described in three independent studies, four in four different studies and one in five independent studies (Table 3A). Three miRNAs were most frequently identified and analysed in the context of fracture healing (Figure 3). miR‐21 has been described as differentially expressed during fracture healing in nine different publications.^45^, ^46^, ^71^, ^86^, ^101^, ^103^, ^109^, ^115^, ^128^ miR‐140‐3p/5p was investigated in seven different reports^48^, ^78^, ^85^, ^103^, ^104^, ^120^, ^129^ and miR‐214‐3p/5p was described in eight different studies.^55^, ^63^, ^81^, ^104^, ^106^, ^111^, ^113^, ^130^

### 3.7 miRNAs in non‐union fractures: clinical screening

Eleven studies performed a screening in patients with non‐union fracture compared to uneventful healing.^53^, ^125^, ^130^, ^131^, ^132^, ^133^, ^134^, ^135^, ^136^, ^137^, ^138^ Blood samples were analysed in *n* = 2^131^, ^137^ studies and callus tissue in *n* = 9^53^, ^74^, ^125^, ^130^, ^132^, ^133^, ^134^, ^135^, ^136^, ^138^ studies (Table 2). The identified miRNAs during screening were then validated in further experiments. Six of these studies^130^, ^131^, ^132^, ^135^, ^136^, ^138^ validated their findings in animal models and in in vitro experiments. Ten studies^53^, ^130^, ^131^, ^132^, ^133^, ^134^, ^135^, ^136^, ^137^, ^138^ only validated the findings in in vitro models, and one study did not perform a cell or animal study.^125^

### 3.8 miRNAs in non‐union fractures: sequencing and microarray analysis

Two out of the 19 studies included in this review performed sequencing,^79^, ^135^ while eight studies^53^, ^125^, ^129^, ^136^, ^137^, ^138^, ^139^, ^140^, ^141^ included a microarray analysis. One study performed RNA sequencing on tissue samples collected from non‐union patients.^135^ After microarray or sequencing, eight studies^53^, ^129^, ^135^, ^136^, ^137^, ^138^, ^140^, ^141^ functionally validated the miRNAs during MSC differentiation in vitro.

One report studied 727 miRNAs at different timepoints during fracture healing process in rat fracture model implementing closed femoral shaft fractures.^139^ A total of 368 miRNAs were identified as upregulated early during fracture healing on day 5 (top four: miR‐339‐3p, miR‐451‐5p, miR‐532‐5p and miR‐551b‐3p), while 207 were increased on day 11 (top four: miR‐221‐3p, miR‐376a‐3p, miR‐379‐3p and miR‐379‐5p). The top four miRNAs for both timepoints were selected for further validation by reverse transcription‐quantitative polymerase chain reaction (RT‐qPCR).^139^ Similarly, Waki et al.^140^ performed microarray analysis on 680 miRNAs from tissue samples harvested on day 14 after surgery to implement a non‐union fracture model or a healing fracture model in rats. They selected five miRNAs, miR‐140‐3p, miR‐140‐5p, miR‐181a‐5p, miR‐181d‐5p and miR‐451a, according to the microarray analysis, for further validation in tissue samples.^140^ Another study performed a microarray analysis on callus tissue samples from patients with identified non‐union fractures 6–8 months after tibial fracture.^125^ miRNAs identified as differentially expressed in non‐union fracture tissue samples were subsequently validated by RT‐qPCR. A similar approach was used by Long et al.,^138^ who compared callus tissue samples of fracture patients and non‐union fracture patients. The authors found 557 miRNAs that were differentially expressed between the two groups and functionally validated the expression of the most differentially expressed miRNA target genes by transfecting BMSCs with miRNA‐mimics or ‐inhibitors.^138^

### 3.9 miRNAs in non‐union fractures: fracture models

The fracture models used were comparable among studies since a femoral fracture model in mice or rats was commonly used. To study non‐union fracture healing, the most common method was cauterisation or removal of the periosteum on the fracture site to avoid normal fracture healing.^129^, ^140^, ^142^, ^143^ Another approach was the creation of a critical size defect.^141^ In this study, the authors used a fracture gap of 1.8 mm for the non‐union fracture model in mice and only 0.25 mm for the normal healing fracture model. A metallic clip was implanted to keep the gap size during the experiment.^141^ One study investigated the effect of DM on the fracture healing process and the miRNAs involved in this disease.^139^ DM was induced by an intraperitoneal injection of 40 mg/kg streptozotocin. Rats with blood glucose levels over 300 mg/dl at 1 week post‐injection were included in the DM group. Non‐DM mice served as a control group. All animals were subjected to femoral shaft fractures.

Out of 14 studies that implemented a fracture model, eight fracture models were performed in rats,^129^, ^132^, ^138^, ^139^, ^140^, ^142^, ^143^, ^144^ and six were performed in mice^130^, ^131^, ^135^, ^141^, ^145^, ^146^ with C57BL/6J being the most common strain.^130^, ^131^, ^136^, ^145^ Two studies did not indicate the mouse strain used.^135^, ^141^

### 3.10 miRNAs in non‐union fractures: in vitro experiments

Fourteen out of 19 studies performed an in vitro experiment to validate clinical or in vivo findings. Five of these studies^130^, ^133^, ^134^, ^136^, ^145^ used osteoblasts, while six studies^53^, ^131^, ^132^, ^135^, ^138^, ^143^ used (B)MSCs with cells cultured under osteogenic conditions. Transfection of MSCs with miRNA‐mimics or ‐inhibitors was also performed.^74^, ^131^ One study used adipose‐derived stem cells (ASCs) to investigate the effect of different miRNAs on the differentiation process.^142^

Even in the case of non‐union‐related studies, a large heterogeneity in cell origins and cell types used was detected. Six studies worked with human primary cells to validate the effect of miRNAs on the differentiation process, which is likely the most clinically relevant experiment.^53^, ^131^, ^132^, ^135^, ^138^, ^142^ Three studies used primary BMSCs/MSCs from rats.^79^, ^143^, ^144^

In total, seven studies used cell lines for in vitro validations. Five studies used the mouse osteoblast cell line MC3T3‐E1,^74^, ^130^, ^133^, ^136^, ^145^ whereas two studies used the human osteosarcoma cell line MG‐63.^134^, ^137^

### 3.11 Summary of validated miRNAs in non‐union fractures

**FIGURE 4:**
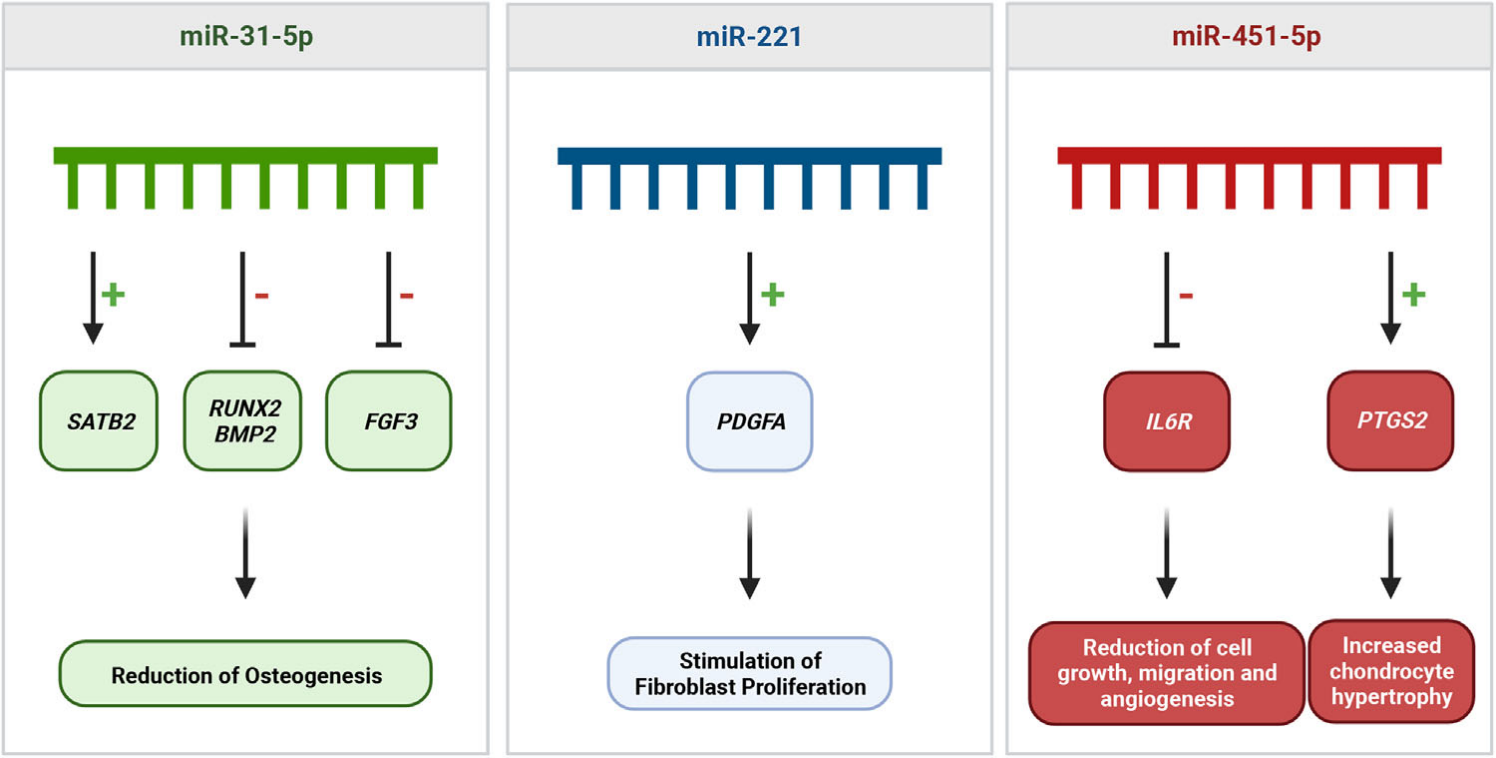
MicroRNAs (miRNAs) in fracture non‐union. Mechanism of action of miR‐31‐5p, miR‐221 and miR‐451‐5p involved in non‐union fractures. ‘+’ indicates promotion, while ‘–’ indicates inhibition of the target gene or pathway. This regulation does not necessarily represent the direct target of the miRNA but rather the overall downstream pathway regulation. Main effect of miR‐31‐5p is the reduction of osteogenesis by inhibition of runt‐related transcription factor 2 (*RUNX2*) and bone morphogenic protein 2 (*BMP2*) expression. miR‐221 leads to increased fibroblast proliferation by upregulation of platelet‐derived growth factor (*PDGFA*). miR‐451‐5p in general decreases cell growth, migration and angiogenesis, all critical factors for fracture healing.

Sixty miRNAs were identified in 18 different studies. Three miRNAs were identified in two different reports: miR‐31‐5p,^140^, ^141^ miR‐221‐3p^134^ and miR‐451‐5p.^134^, ^139^, ^140^ miR‐140‐5p was reported by three independent studies^129^, ^141^, ^142^ (Table 3B). Three of the most common miRNAs involved in disturbed fracture healing including known targets are depicted in Figure 4.

## 4 DISCUSSION

This review aimed to systematically summarise findings from clinical populations, animals and cell models to identify miRNAs with the potential to be used as biomarkers to monitor the fracture healing process. Our approach identified miR‐21, miR‐140 and miR‐214 as potential biomarkers for fracture healing in general (Figure 3), while miR‐31‐5p, miR‐221 and miR‐451‐5p have a potential in the monitoring of fracture healing and non‐union fractures (Figure 4). The discussion is focused on those six miRNAs, as they have been validated in several studies. In addition, their targets and the known regulated pathways linked to bone healing are presented, further supporting their promising role as biomarkers.

### 4.1 miRNAs in fracture healing: miR‐21

miR‐21 was identified in a microarray study as differentially expressed in osteoporotic compared to non‐osteoporotic patient samples.^45^ Nine independent studies^45^, ^46^, ^71^, ^86^, ^101^, ^103^, ^109^, ^115^, ^128^ provided evidence for a pivotal role of miR‐21 in rat and mouse fracture models. Treatment of the fracture site with miR‐21 mimics increased callus formation and induced bridging of the fracture gap compared to an injection of scrambled miRNA.^46^ In addition, overexpression of miR‐21 in MSCs promoted osteogenesis and accelerated bone fracture healing,^101^ consistent with the finding that miR‐21 is involved in early stages of intramembranous ossification as an important step in fracture healing.^128^ miR‐21 promotes fracture healing by activating the phosphoinositid 3‐kinase/serine‐threonine‐kinase/RAC‐alpha serine/threonine‐protein kinase (PI3K/AKT) signalling pathway,^109^ which regulates a number of processes, such as cell survival, proliferation, growth, metabolism and angiogenesis.^147^ Controversially, Sheng et al.^115^ showed that miR‐21 activates the extracellular signal‐related kinase pathway concluding that a downregulation of miR‐21 promotes fracture healing in rats. In addition, miR‐21 regulates the TGF‐β pathway by targeting the phosphatase and tensin homologue deleted on chromosome 10 (*PTEN*)^148^ and by targeting the TGF‐β‐receptor 2 (*TGFBR2*).^149^ Furthermore, miR‐21 facilitates osteoclastogenesis by inhibiting programmed cell death 4 (*PDCD4*),^150^ inhibits cell migration and promotes cytoskeletal organisation. The members of dedicator of cytokinesis (DOCK) 180‐related protein superfamily have also been identified as target genes.^151^ Downregulation of DOCK inhibits cell migration and promotes cytoskeletal organisation.

### 4.2 miRNAs in fracture healing: miR‐140

miR‐140 has been shown to promote osteogenic differentiation and calcium deposition in bone^120^ and to play a role in skeletal development by regulating endochondral ossification.^152^ Its role has been validated in a mouse fracture model where it promoted fracture healing after injection in the fracture site.^120^ Waki et al.^129^ identified miR‐140 as differentially expressed in a screening of fracture patients with healed fractures, compared to non‐healed fractures. Orth et al.^141^ confirmed that miR‐140‐3p and miR‐140‐5p were significantly downregulated in a non‐union mouse fracture model. These observations may be explained by involvement of miR‐140‐3p in activation of the Wnt signalling pathway, which promotes osteogenesis^153^ and the fracture healing process.^48^ miR‐140 may also control fracture healing by regulating the expression of Toll‐like receptor 4 (*TLR4*) and *BMP2*.^103^, ^142^ *Dnpep* was identified as a target gene of miR‐140‐5p^141^, ^152^ and an upregulation of *Dnpep* caused skeletal defects by inhibiting BMP signalling.^152^ However, Orth et al.^141^ also predicted an inhibitory effect of miR‐140 on BMP2 through the downregulation of stromal cell‐derived factor 1α (SDF‐1α) (*CXCL12*).

In contrast to the reported positive effects of miR‐140 in fracture healing, it was also shown that the inhibition of miR‐140‐5p promoted osteogenesis in ASCs and enhanced fracture healing in an atrophic non‐union rat model.^142^ A transfection of chondrocytes with miR‐140 mimics increased *SMAD1* expression and suppressed the hypertrophy of chondrocytes by controlling the BMP pathway.^154^

The role of miR‐140 during fracture healing is still not completely understood. In particular, it is unclear which outcome is promoted by an increased miR‐140 level at an early timepoint after a fracture. However, it is likely that the regulation of miR‐140 during fracture healing process is time‐dependent or the different strands are regulated differently.

### 4.3 miRNAs in fracture healing: miR‐214

A review of the literature indicates contrasting findings regarding the role of miR‐214 in fracture healing delay or non‐union. Screening of fragility fracture patients showed decreased miR‐214 levels in blood and tissue samples, and miR‐214 was shown to regulate proliferation and apoptosis of osteoblasts and bone formation by inhibiting the expression of SRY‐box transcription factor 4 (*SOX4*).^55^ miR‐214 was increased in patients directly following a fracture and inhibition of miR‐214 promoted cell survival and extracellular matrix formation in the early phase of fracture healing by targeting type IV collagen (*COL4A1*),^63^ a component of basement membranes.^155^ Upregulated miR‐214 led to improved fracture healing by regulating the BMP/Smad signalling pathway.^111^ miR‐214 is also involved in the modulation of Wnt/β‐catenin pathway and inhibits endochondral ossification, which led to delayed fracture healing in fracture patients.^113^ Functional studies suggested that suppressing miR‐214 in MSCs enhances *RUNX2* levels and promotes osteogenic differentiation. Li et al.^106^ identified *CTNNB1* as a target of miR‐214, which is relevant since *CTNNB1* encodes β‐catenin, a mediator in the Wnt signalling pathway that activates the osteogenic transcription factor RUNX2. Thus, miR‐214 may function as a possible therapeutic target to improve the fracture healing process and should be further investigated to decrease the risk for delayed fracture healing or non‐union.

### 4.4 miRNAs in non‐union fractures

Compared to investigations on miRNAs involved in physiological bone healing processes, data on miRNAs involved in non‐union fractures are scarce. Nonetheless, some miRNAs have been identified and validated for impaired bone healing. Table 2 includes miRNAs that have been described as differentially expressed during non‐union fractures. Of note, miR‐31‐5p, miR‐221 and miR‐451‐5p have not been reported in studies with undisturbed fracture healing and may thus be specifically regulated in non‐union fractures and may represent promising miRNAs with biomarker potential. Those miRNAs should be further validated to examine their exact role during the fracture healing process.

### 4.5 miRNAs in non‐union fractures: miR‐31a‐5p

Profiling miRNAs in rat non‐union fractures showed an upregulation of miR‐31a‐3p/‐5p.^140^ miR‐31a‐3p/‐5p were among the most upregulated miRNAs in the non‐union fracture group compared to healing fractures on post‐fracture day 14. Orth et al.^141^ reported a comparable observation from a mouse non‐union model with a 1.8 mm femoral fracture gap, suggesting that miR‐31‐5p is a negative regulator of MSC osteogenic differentiation.^156^ Accordingly, miR‐31 was reported to target the expression of special AT‐rich sequence‐binding (SATB) homeobox 2 gene (*SATB2*),^156^ which encodes a pro‐osteoblastogenic transcription factor.^157^ In addition, *RUNX2* and *BMP2* are known targets of miR‐31a‐5p and inhibition of miR‐31a‐5p promoted the osteogenic differentiation of MSCs in vitro.^158^

### 4.6 miRNAs in non‐union fractures: miR‐221

Microarray analysis of callus tissue samples from atrophic non‐union fracture patients revealed an increased expression of miR‐221,^53^ and functional studies in MSCs provided evidence that miR‐221 overexpression inhibits the expression of PDGF subunit A (PDGFA).^53^ This is of relevance since inhibition of PDGFA stimulates fibroblast proliferation in the early stages of fracture healing leading to improved bone formation.^159^ Using a diabetic rat model of delayed fracture healing and non‐union, Takahara et al.^139^ also observed an altered expression level of miR‐221‐3p, which was highly upregulated on post‐fracture days 5, 7 and 11 in the DM group.

### 4.7 miRNAs in non‐union fractures: miR‐451‐5p

miR‐451‐5p was identified using microarray analysis in a rat DM model after analysing callus samples from the fracture site indicating elevated levels from days 5 to 14 after a surgery‐implemented fracture.^139^ Functional studies showed that miR‐451‐5p inhibits cell growth, migration and angiogenesis via downregulation of *IL6R*.^160^ However, in a fracture model implemented in non‐diabetic rats, a stronger upregulation of miR‐451‐5p in standard healing fractures was observed in comparison to non‐union fractures.^129^ Furthermore, upregulation of miR‐451‐5p increased cyclooxygenase 2 (COX2) protein levels linking miR‐451 to endochondral ossification by increased chondrocyte hypertrophy.^161^, ^162^

### 4.8 miRNAs in osteoporotic fractures

Even though this was not a category that was systematically analysed within this review, some miRNAs associated with osteoporotic fractures have also been identified. Patients suffering from osteoporosis have a higher incidence of non‐traumatic fractures and have an elevated risk for non‐union. The internal fixation to treat fractures in osteoporotic patients suffers from insufficient strength and stability because of low BMD.^163^ Nine of the included studies^46^, ^47^, ^81^, ^102^, ^104^, ^106^, ^111^, ^164^ implemented an osteoporotic fracture model to focus on this group of patients. miR‐140, miR‐214, miR‐21 and miR‐26a were shown to improve osteogenic differentiation in osteoporotic bone defects.^46^, ^102^, ^130^ miR‐22 stimulated osteogenic differentiation by inhibition of MYC proto‐oncogene (MYC)/PI3K/AKT pathway and was beneficial in an osteoporotic bone model to promote bone healing.^47^ Possible evidence for a role of miR‐214 in fracture healing in an ovariectomised mouse model was reported by Wang et al.,^81^ who transfected HUVECs with a miR‐214 inhibitor, leading to improved tube formation and cell migration. Mechanical stimulation across the fracture gap in an osteoporotic mouse model decreased miR‐214 levels improving fracture healing. In addition, miR‐214 suppression enhanced *RUNX2* expression and promoted osteogenic differentiation in an osteoporotic rat model,^106^ while enhanced miR‐214 expression delayed healing of osteoporotic model fractures likely by inhibiting the BMP/Smad signalling pathway.^111^

In terms of improved fracture healing in osteoporosis, miR‐187‐induced osteogenic differentiation, bone reconstruction and healing in a mouse osteoporotic fracture model.^65^ A subsequent analysis in a model of ovariectomy to induce osteoporosis confirmed the importance of miR‐187.^65^

It has also been suggested that knockdown of miR‐100 can improve the osteogenic differentiation of BMSCs by promoting the AKT/mechanistic target of rapamycin kinase (mTOR) pathway.^164^

The identified miRNAs may be used to predict the risk for a non‐union in osteoporotic fracture patients and to improve the treatment of fragility fractures due to a low BMD.

### 4.9 Future perspectives

Our analyses suggest that specific miRNAs with biomarker potential exist, which may be used to predict disturbed fracture healing alone or in combination. However, translation into clinical practice requires a standardised approach for future studies, also with respect to minimally invasive sampling procedures such as liquid biopsies. Also, it needs additional evidence that data from the osteoporotic models are relevant in humans with osteoporosis. Since there is a lack of knowledge concerning the comparability of local versus circulating miRNA levels, we suggest combined analysis of the herewith identified miRNAs in non‐union fracture patients’ callus tissue collected during revision and concurrent blood sampling. Correlation with clinical outcomes should be performed over at least 6 months after fracture. Additional individual factors with influence on miRNA levels should be thoroughly investigated and reported, including age, DM, menopausal state and *T*‐score, in the case of osteoporotic patients. Further research including functional analysis of identified miRNAs using established in vivo or in vitro models is needed to identify the involved target molecules and pathways and to understand miRNA expression profiles over time. The overall vision is to use these miRNAs as diagnostic markers, alone or in combination with other factors, to predict the risk of fracture healing disturbances and improve the decision making and the individual treatment options for the patients. Moreover, these miRNAs or the pathways they affect also represent promising therapeutic targets to improve fracture healing.

## 5 CONCLUSIONS

Despite large heterogeneity in the field of miRNAs and fracture healing, this systematic analysis of clinical screenings and functional validation studies revealed a set of miRNAs with biomarker potential in disturbed fracture healing. Based on our investigation, miR‐31‐5p, miR‐221 and miR‐451‐5p appear to be involved in processes linked to fracture non‐union and could be used to predict disturbed bone healing. For fracture healing in general, focusing on miR‐21, miR‐140 and miR‐214 is promising for future investigations. Further studies will have to focus on those miRNAs and on their further validation during fracture healing before miRNA‐based theranostic approaches become an option.

## CONFLICT OF INTEREST

The authors declare they have no conflicts of interest.
